# Two Antimicrobial Heterodimeric Tetrahydroxanthones with a 7,7′-Linkage from Mangrove Endophytic Fungus *Aspergillus flavus* QQYZ

**DOI:** 10.3390/molecules27092691

**Published:** 2022-04-22

**Authors:** Zhenming Zang, Wencong Yang, Hui Cui, Runlin Cai, Chunyuan Li, Ge Zou, Bo Wang, Zhigang She

**Affiliations:** 1 School of Chemistry, Sun Yat-sen University, Guangzhou 510275, China; zangzhm@mail2.sysu.edu.cn (Z.Z.); yangwc6@mail2.sysu.edu.cn (W.Y.); zoug5@mail2.sysu.edu.cn (G.Z.); 2 School of Pharmaceutical Sciences, Guangzhou University of Chinese Medicine, Guangzhou 510006, China; cuihui@gzucm.edu.cn; 3 College of Science, Shantou University, Shantou 515063, China; rlcai@stu.edu.cn; 4 College of Materials and Energy, South China Agricultural University, Guangzhou 510642, China; chunyuanli@scau.edu.cn

**Keywords:** tetrahydroxanthone dimer, mangrove endophytic fungus, antifungal activities, antibacterial activities

## Abstract

Mangrove endophytic fungi represent significant and sustainable sources of novel metabolites with unique structures and excellent biological activities, attracting extensive chemical investigations. In this research, two novel heterodimeric tetrahydroxanthones, aflaxanthones A (**1**) and B (**2**), dimerized via an unprecedented 7,7′-linkage, a sp^3^-sp^3^ dimeric manner, were isolated from the mangrove endophytic fungus *Aspergillus flavus* QQYZ. Their structures were elucidated through high resolution electrospray ionization mass spectroscopy (HRESIMS) and nuclear magnetic resonance (NMR) spectroscopy, the absolute configurations of them were determined by a single-crystal X-ray diffraction combined with calculated electronic circular dichroism (ECD) spectra and a 1D potential energy scan. These compounds were evaluated for antifungal activities in vitro and exhibited broad-spectrum and potential antifungal activities against several pathogenic fungi with minimum inhibitory concentration (MIC) values in the range of 3.13–50 μM. They also performed moderate antibacterial activities against several bacteria with MIC values in the range of 12.5–25 μM. This research enriched the resources of lead compounds and templates for marine-derived antimicrobial drugs.

## 1. Introduction

Xanthones are a type of polyketides of dibenzo-γ-pyrone structures with structural diversity and wide distribution; they are mainly found in plants, fungi, and lichens [1,2,3]. Due to the different degrees of oxidation, the distinctions of substituent positions and polymerization, the diverse structures of xanthones lead to a broad spectrum of promising biological activities, such as *α*-glucosidase inhibitory [4,5], cytotoxicity [6,7,8,9], pancreatic lipase inhibitory [10], anti-inflammatory [9,11,12], antibacterial [13,14], antifungal [15,16,17], antiosteoporosis [18], pro-apoptotic, and immunostimulatory [19] activities. Xanthone dimers from fungi often process biaryl bonds as the major linkage methods, generally with 2,2′, 2,4′, and 4,4′-biaryl bonds [2], and rarely in the form of ether bonds, C3-N-C2′ bridge, heptacyclic 6/6/6/5/6/6/6, or the 6/6/6/6/6/6/6 ring system [13,20,21,22]. Mangrove endophytic fungi, representing considerable resources of novel metabolites, produce many unique xanthone dimers, such as incarxanthone F from *Peniophora incarnata* Z4, which is dimerized via a C3-N-C2′ bridge [20], phomoxanthones C–E with a 2,2′-biaryl bond isolated from *Phomopsis* sp. xy21 [23], penicillixanthone B from *Setophoma terrestris* (MSX45109) containing a 2-4′-linkage [14], and deacetylphomoxanthone C with a 4,4′-linkage produced by *Phomopsis* sp. HNY29-2B [7].

**Figure 1:**
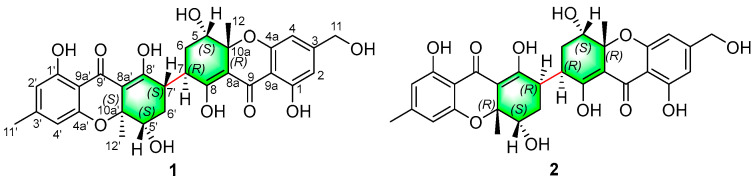
Structures of compounds **1** and **2**.

Previously, a series of novel bioactive metabolites were characterized from mangrove endophytic fungi in the South China Sea, in our group [24,25,26,27,28,29]; during ongoing research, a chemical investigation was performed on an endophytic fungus, *Aspergillus flavus* QQYZ isolated from a fresh blade of the mangrove plant *Kandelia candel*, collected from Huizhou in Guangdong province. Two new heterodimeric tetrahydroxanthones, aflaxanthones A (**1**) and B (**2**), which were dimerized via an unprecedented 7,7′-linkage, a non-biaryl dimeric manner, were obtained from the culture broth of the fungus (Figure 1). It represents the first report of xanthone dimers formed by a non-aromatic single bond connection. In the antimicrobial activities assays, these new compounds have exhibited broad-spectrum and pronounced antifungal and antibacterial activities. Herein, we report the isolation, structure elucidation, and antimicrobial activities of the two compounds.

## 2. Results

After 28 days of cultivation on rice solid medium, the EtOAc extract of fungus *Aspergillus flavus* QQYZ was fractionated with column chromatography, using silica gel and Sephadex LH-20, followed by chiral high performance liquid chromatography (HPLC) for the two novel compounds from a mixed component.

**Figure 2:**
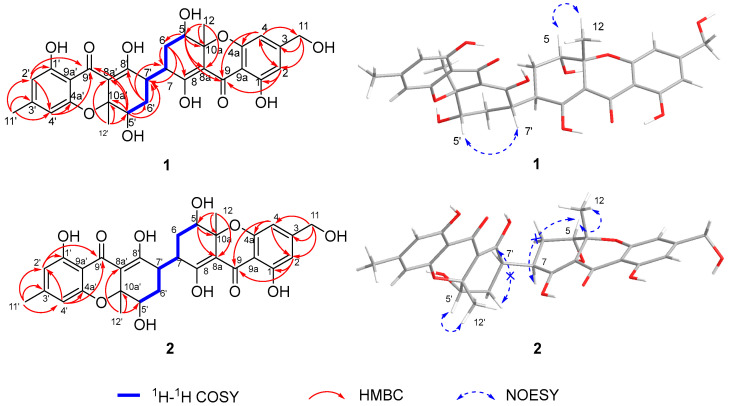
Key ^1^H-^1^H COSY, HMBC and NOESY interactions of **1** and **2**.

Aflaxanthone A (**1**) was obtained as a yellow powder with the molecular formula C_30_H_30_O_11_ deduced from the HRESIMS peaks at 567.1866 [M + H]^+^ (calcd for C_30_H_31_O_11_, 567.1861), corresponding to 16 degrees of unsaturation. The UV spectrum and the NMR spectra of **1** indicated a characteristic of a xanthone dimer derivative [13]. The ^1^H NMR spectroscopic data, performed in a mixed solution of MeOD-*d*_4_ and CDCl_3_ of **1** (Table 1), exhibited four aromatic singlet protons at *δ*_H_ 6.45 (s, H-2), 6.42 (s, H-4), 6.27 (s, H-2′), and 6.26 (s, H-4′), one hydroxymethyl at *δ*_H_ 4.51 (s, H_2_-11), two methylene at *δ*_H_ 2.10–2.02 and 1.71–1.62 (m and m, H_2_-6), 2.20–2.14 and 1.87–1.79 (m and m, H_2_-6′), four methines at *δ*_H_ 4.20 (dd, *J* = 12.4, 4.2 Hz, H-5), 4.12 (dd, *J* = 4.3, 1.9 Hz, H-5′), 3.44–3.35 (m, H-7), and 3.44–3.35 (m, H-7′), and three methyl at *δ*_H_ 2.25 (s, H_3_-11′), 1.48 (s, H_3_-12), 1.45 (s, H_3_-12′). Notably, unlike xanthone dimers that are typically dimerized through a biaryl bond, compound **1** had four aromatic protons instead of two [2,7,23]. Moreover, the hydroxymethyl at *δ*_H_ 4.51 ppm and methyl at *δ*_H_ 2.25 ppm, combined with the molecular formula, can exclude the linkage of an ether bond compared with asperdichrome and 5-*epi*-asperdichrome [22,30]. Thus, there might be a new linkage of dimerization. Combined with the HSQC, there were 18 sp^2^-hybridized carbons and 12 sp^3^ carbons in the ^13^C NMR spectrum, including two ketone carbonyls (*δ*_C_ 188.6 and 188.4), two enolic fragments (*δ*_C_ 176.9, 107.8, 175.0, and 105.8), twelve aromatic carbon atoms (δ_C_ 162.4, 162.2, 159.3, 159.3, 153.6, 150.8, 110.7, 109.8, 107.6, 106.6, 106.1, and 105.3), two quaternary carbons (*δ*_C_ 81.8 and 81.5), four methines (*δ*_C_ 71.8, 70.8, 39.9, and 37.1), two methylenes (*δ*_C_ 29.9 and 28.0), one hydroxymethyl (*δ*_C_ 64.1), and three methyl carbons (*δ*_C_ 26.1, 22.5, and 20.1). The NMR signals of **1** appeared duplication but not overlapping; moreover, C-11 (*δ*_H_ 4.51, *δ*_C_ 64.1) and C-11′ (*δ*_H_ 2.25, *δ*_C_ 22.5) exhibited in a single-handed form, meaning a heterodimeric skeleton with a minute difference of 3-methyl and 3-hydroxymethyl between the planar structure of the two units. The heteronuclear multiple bond correlation (HMBC) from H-2 to C-1, C-4, C-9, and C11, from H-4 to C-2, C-4a, C-9, and C-11, from H-11 to C-2, C-3, and C-4, implied the presence of a 1,2,4,6-tetrasubstituted benzene system (Figure 2). The ^1^H-^1^H homonuclear chemical shift correlation spectroscopy (COSY) cross-peak of H-5/H-6 and H-6/H-7, along with the HMBC correlations from H-5 to C-6, C-7, C-8a, C-10a, and C-12, from H-12 to C-5, C-8a, and C-10a, from H-6 to C-7, C-8, and C-7′ illustrated a 1,2,3,4,6-pentasubstituted cyclohexene moiety. The aforementioned ketone groups, benzene and cyclohexene rings in two units, accounted for 14 degrees of unsaturation, the remaining 2 degrees of unsaturation in conjunction with a further HMBC correlation from H-5 to C-9 and the chemical shift of C-4a and C-10a indicated the linkage of the two moieties via C8a-C9/C8a’-C9′ and C4a-O-C10a/C4a′-O-C10a′ to form a tetrahydroxanthone scaffold. As for the connection of two fragments, both benzene ring fragments had two proton signals, indicating that the dimerization mode was different from the common mode of a biaryl bond [7,14,23]. However, the 7/7′-methines and the HMBC correlations from H-6 to C-7′, from H-6′ to C-7, indicated the two units dimerized via a unique 7,7′-bond that may have been discovered in xanthone dimers for the first time, which was later proved by the X-ray diffraction experiment.

**Figure 3:**
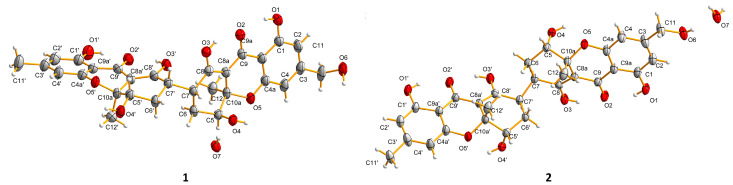
X-ray ORTEP drawing of **1** and **2**.

**Figure 4:**
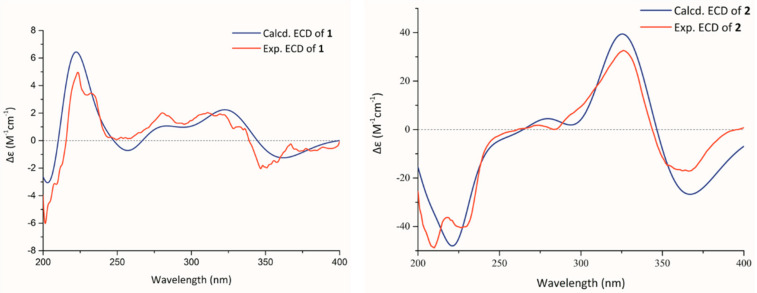
Experimental ECD spectra in acetonitrile and calculated ECD spectra at B3LYP/6–311+g (d,p) level of **1** and **2**.

The relative configuration of **1** can be revealed by the nuclear overhauser effect spectroscopy (NOESY) spectrogram (Figure 2), as the key correlations of H-5 with H_3_-12 suggested that H-5 and H_3_-12 positioned on the same face and H-7 on the other face. The NOESY correlation of H-5′ with H-7′ implied H-5′ and H-7′ had the same orientation and the H_3_-12′ was in the opposite direction. The above NOESY correlations showed different chirality of the two units at aliphatic parts of C-5/C-5′, C-7/C-7′, and C-10a/C-10a’. Fortunately, the single crystals of **1** were crystallized slowly in the mixture of methanol and dichloromethane with a same volume ratio. Thus, the absolute configuration of **1** was confirmed by the following X-ray diffraction analysis using the Cu K*α* radiation with a flack parameter of 0.02(13) (Figure 3). Meanwhile, to further verify the stereo structure, ECD calculations were carried out by the time-dependent density functional theory (TDDFT) approach at B3LYP/6–311+g (d,p) level [31], using the single-crystal stereochemical structure as input. The results of the theoretical ECD spectra basically showed a consistent Cotton effect with the experimental curve in acetonitrile (Figure 4). Consequently, the absolute configuration of compound **1** was determined as 5*S*,7*R*,10a*R*,5′*S*,7′*S*,10a’*S*. The results of a single crystal diffraction analysis and theoretical ECD spectra strongly proved that this was the first discovery of a xanthone dimer connected by a 7-7′ linkage of a non-biaryl bond.

Aflaxanthone B (**2**) was also acquired as a yellow powder, sharing the same molecular formula of C_30_H_30_O_11_ as **1,** established by HRESIMS data at *m*/*z* 589.1676 ([M + Na]^+^, calcd for C_30_H_30_O_11_Na, 589.1680). Since showing the same UV data as **1**, it suggested that **2** was also a tetrahydroxanthone derivative. To deduce the structure of **2**, the NMR experiments were implemented at room temperature, 298 K. However, confusingly, in the ^1^H NMR spectrum of **2**, the aliphatic protons of H-5/5′, H_2_-6/6′ and H-7/7′ were unable to be observed contrasted with **1** (Table 1). The same phenomenon appeared in the ^13^C NMR spectrum, as the carbon signals of C-5/5′, C-6/6′, C-7/7′, C-8/8′, and C-8a/8a’ were absent, the signals of the connection moiety between the two units disappeared, then the low-temperature ^1^H NMR experiments were preliminarily conducted under 273 and 243 K to clarify the integral structure of **2** [31,32,33]. Broad peaks at 273 K and obvious resonance peaks at 243 K emerged (Figures S12 and S13), signifying the NMR experiments at 243 K can be feasible; meanwhile, signals in the low field of ^13^C NMR were separating at 243 K, indicating rotational isomers existed in **2** [34]. Further comprehensive analysis of the new set of NMR spectra (Table 1) experiments performed at 243 K with clear carbon peaks and correlations of the 2D NMR spectrum, especially a striking key ^1^H-^1^H COSY cross-peak of H-7/H-7′ (Figure 2), showed the same planar structure as **1** dimerized with a 7,7′-linkage. The key NOESY correlations of H-5 with H-12 and H-5′ with H-12′ and the deficiency of interactions of H-7 with H-5 and H-7′ with H-5′ evinced the same central chirality of the two units (Figure 2). The single crystal of **2** was obtained under the same conditions as **1**, and the subsequent X-ray diffraction experiment confirmed the absolute configuration of **2** to be 5*S*,7*R*,10a*R*,5′*S*,7′*R*,10a’*R* (Figure 3), giving a flack parameter of −0.08 (5). Owing to the asymmetry by the 3-hydroxymethyl and the 3′-methyl, but with the same appearing ratio, a disorder existed in the X-ray diffraction [35]. Then the analogy was made between the theoretical and experimental ECD spectra by the same approach as **1**, and consistent Cotton effects of experimental and calculated curves were observed (Figure 4). Ultimately, the absolute configuration of **2** was unambiguously elucidated as 5*S*,7*R*,10a*R*,5′*S*,7′*R*,10a’*R*; it was also proved to process the unique 7-7′-linkage.

**Figure 5:**
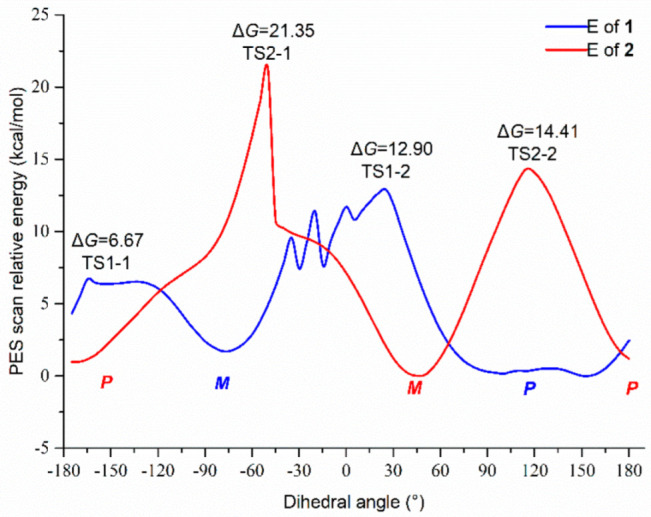
The 1D PES scans on the dihedral angle C8-C7-C7′-C8′ at B3LYP/6–31 g (d,p) level of **1** and **2**.

A portion of the NMR signal around the chiral axis could broaden or disappear in some axial chiral compounds [32,34,36]; in order to verify the presence of the axial chirality in **2**, a 1D potential energy scan (PES) was conducted on the dihedral angle C8-C7-C7′-C8′ by modredundant optimization using the DFT method at the B3LYP/6–31 g (d,p) level in Gaussian 09 [31] to calculate the rotational energy barrier around the C7-C7′ bond (Figure 5) [19]. The relative Gibbs energy barriers at each transition state (TS) for the *M*/*P* conversion were 21.35 kcal/mol (TS2-1) and 14.41 kcal/mol (TS2-2), indicating the coalescence of the *M/P* isomer in **2** at room temperature [31,37]. On account of the interconversion in **2**, the absence of the NMR signals around the C7-C7′ bond at 298 K occurred, and the ^13^C NMR spectrum showed separated signals of the chromone moieties at 243 K simultaneously, suggesting the 8-OH and 8′-OH cannot impose sufficient spatial hindrances to interrupt the free rotation of the two units [36,38]. As an additional supplement, we also calculated the Gibbs energy barrier of **1**; the results were 6.67 kcal/mol (TS1-1) and 12.90 kcal/mol (TS1-2), which meant the two units of **1** could rotate freely and **1** could be developed as a single compound [32], and a complete set of NMR signals of **1** at room temperature could be observed as proof. Hence, the two compounds can be determined as optically pure monomers [16,20]. Moreover, the results above could mean that there might also be axial chirality around the sp^3^–sp^3^ hybrid carbon–carbon bond. If the space occupations of the groups on both sides of the axis are large enough and the signals in NMR change, it may be necessary to verify the existence of axial chirality.

Compounds **1** and **2** were evaluated for antifungal activities against *Candida albicans* and four agricultural plant pathogenic fungi—*Fusarium oxysporum*, *Penicillium italicum*, *Collettrichum musae*, and *Colletotrichum gloeosporioides*; **1** showed promising inhibitory activity against *C.* *gloeosporioides*, for 3.13 μM, while the MIC of the positive control ketoconazole was 0.1 μM, and moderate activity against *F. oxysporum* and *C. albicans* with MIC was 12.5 μM; **2** exhibited moderate activity against *F. oxysporum* and *C. musae* with MIC 12.5 μM, respectively (Table 2). As for antibacterial activities against methicillin-resistant *Staphylococcus aureus* (MRSA), *Pseudomonas aeruginosa*, and *Bacillus subtilis*, compound **1** possessed moderate inhibitory activity against MRSA with a MIC value 12.5 μM, and the two compounds had moderate inhibitory effects on *B. subtilis* with MIC 25 μM compared to the positive control ampicillin with MIC 0.39 and 0.39 μM against MRSA and *B. subtilis*, respectively (Table 2). The experiments show that the compounds could be used as spectral antifungal and antibacterial drug precursors for more in-depth research.

## 3. Materials and Methods

### 3.1. General Experimental Procedures

The melting points were measured on a SGW X-4B micromelting point apparatus ((Shanghai Precision Scientific Instrument Co., Ltd, Shanghai, China) and were uncorrected. Optical rotations were measured on MCP 300 (Anton Paar, Graz, Austria) polarimeter at 25 °C. ECD spectra were obtained on Chirascan CD spectrometer (Applied Photophysics, London, UK). UV data were recorded on TU-1900 spectrophotometer (Presee, Beijing, China) in acetonitrile solution. IR spectra were obtained on Frontier FTIR (PerkinElmer, Waltham, MA, USA) spectrometer using the ATR method. NMR experiments were recorded on Bruker Avance 500 spectrometers (500 and 125 MHz) and (Bruker Biospin, Switzerland) the solvent signals of MeOD-*d*_4_ (*δ*_C_ 49.0/*δ*_H_ 3.31 ppm) were used as references. HRESIMS data were acquired on a Thermo Fisher LTQ Orbitrap Elite high-resolution mass spectrometer (Thermo Fisher Scientific, Waltham, MA, USA), respectively. Column chromatography (CC) was carried out on silica gel (200−300 mesh, Qingdao Marine Chemical Factory, China), and Sephadex LH-20 (GE Healthcare Bio-Sciences AB, Stockholm, Sweden). Semi-preparative HPLC was performed on at Thermo U3000 HPLC system using a Daicel Chiralcel OD-H column (4.6 × 250 mm, 5 μm, Daicel, Japan) and the detection wavelengths were 215, 254, 280, and 332 nm. X-ray crystallographic analyses were conducted on an Agilent Gemini Ultra diffractometer (Cu K*α* radiation, Agilent, Oxfordshire, UK).

### 3.2. Fungal Material

The fungus strain *Aspergillus flavus* QQYZ in this work was isolated from a fresh blade of the mangrove plant *Kandelia candel*, collected from Huizhou in Guangdong province, China, and the strain was numbered as QQYZ. The fungal strain was identified by its sequence of the internal transcribed spacer (ITS) analysis of rDNA, and the following BLAST search result showed it was most similar (98%) to the strain *Aspergillus flavus* (compared to JQ776536.1). The sequence data of the fungus were submitted to GenBank, accession no. OK655763. The fungus is stored in our laboratory at Sun Yat-sen University with cryopreservation tubes at −20 °C.

Pathogenic bacteria, including methicillin–resistant *Staphylococcus aureus* (A7983, clinical isolate contributed by Prof. Yu in the Dalian Friendship Hospital, Dalian, China), *Pseudomonas aeruginosa* (ATCC 9027), *Bacillus subtilis* (ATCC 6633), pathogenic fungi involving *Candida albicans* (ATCC 10231), *Fusarium oxysporum*, *Penicillium italicum*, *Collettrichum musae*, and *Colletotrichum gloeosporioides* (contributed by Prof. Li at the College of Material and Energy, South China Agricultural University) were used in the antimicrobial tests.

### 3.3. Fermentation and Isolation

The fungus was cultured on rice solid medium in 60 Erlenmeyer flasks with a volume of 1 L each, containing 50 g rice and 50 g 0.3% brine, for 28 days after 5-day proliferation in 0.6 L potato dextrose broth. Then the culture medium and the mycothallus were soaked with MeOH and extracted with EtOAc after concentrations to 40 g of crude extract. The crude extract was separated by a silica gel column using petroleum ether and ethyl acetate with a ratio from 1:0 to 0:1 in 10 fractions (F1 to F10). Fraction F5 (1.93 g) was eluted on Sephadex LH-20 using CH_2_Cl_2_ and MeOH (1:1 by volume) to yield three sub-fractions, and the second sub-fraction was separated by normal phase HPLC with isopropanol and *n*-hexane (35:65 by volume) to afford compound **1** (8.5 mg, *t*_R_ 12.5 min) and compound **2** (12.1 mg, *t*_R_ 8.0 min) (Figure S19).

Aflaxanthone A (**1**): yellow powder, mp 179–182 °C; [*α*]${}_{D}^{25}$ +17.0 (*c* 0.03, CH_3_CN); UV (CH_3_CN) *λ*_max_ (log *ε*) 210 (1.6), 282 (0.3), 350 (1.7) nm; ECD (CH_3_CN) *λ*_max_ (Δ *ε*) 201 (−6.27), 223 (+4.96), 281 (+2.00), 311 (+2.05), 347 (−2.14); IR *ν*_max_ 3367, 2922, 1641, 1603, 1574, 1455, 1361, 1296, 1233, 1200, 1083, 1047, 882 cm^−1^; HRESIMS *m*/*z* 567.1866 [M + H]^+^ (calcd for C_30_H_31_O_11_, 567.1861); ^1^H NMR (MeOD-*d*_4_ and CDCl_3_, 500 MHz), and ^13^C NMR (MeOD-*d*_4_ and CDCl_3_, 125 MHz) in Table 1.

Aflaxanthone B (**2**): yellow powder, mp 175–177 °C; [*α*]${}_{D}^{25}$ −60.6 (*c* 0.03, CH_3_CN); UV (CH_3_CN) *λ*_max_ (log *ε*) 210 (1.5), 282 (0.3), 349 (1.6) nm; ECD (CH_3_CN) *λ*_max_ (Δ *ε*) 210 (−48.83), 227 (−40.46), 326 (+32.63), 362 (−17.00); IR *ν*_max_ 3395, 2923, 1643, 1607, 1578, 1459, 1367, 1299, 1234, 1202, 1084, 1055, 846 cm^−1^; HRESIMS *m*/*z* 589.1676 [M + Na]^+^ (calcd for C_30_H_30_O_11_Na, 589.1680); ^1^H NMR (MeOD-*d*_4_ and CDCl_3_, 500 MHz), and ^13^C NMR (MeOD-*d*_4_ and CDCl_3_, 125 MHz) in Table 1.

### 3.4. X-ray Crystallographic Analysis of Compounds ***1*** and ***2***

The single crystal of compounds **1** and **2** were acquired from a mixed solution of equal volumes of MeOH and CH_2_Cl_2_. The data were recorded by Agilent Xcalibur Nova single-crystal diffractometer using Cu K*α* radiation (*λ* = 1.5418 Å). The structures were solved by direct methods with the SHELXT software and refined by full-matrix least squares calculations. Non-hydrogen atoms were refined by anisotropic displacement parameters and hydrogen atoms were located on the calculated positions. The crystallographic data of **1** and **2** were deposited to the Cambridge Crystallographic Data Centre.

Crystallographic data for **1**: C_30_H_30_O_11_·H_2_O, *M*_r_ = 584.55, tetragonal, space group: *I*4_1_, *Z* = 8, *a* = 18.7672 (3) Å, *b* = 18.7672 (3) Å, *c* = 17.2209 (3) Å, *α* = *β* = *γ* = 90°, *V* = 6065.3 (2) Å^3^, *D*_c_ = 1.280 g/cm^3^, *μ* = 0.840 mm^−1^, *T* = 100 K, *F*(000) = 2464. Crystal size: 0.27 × 0.12 × 0.07 mm^3^, 15,965 reflections were collected (6.660° < 2*θ* < 149.146°), and 6020 independent reflections (*R*_int_ = 0.0418) were used in all calculations. The final *R*_1_ value was 0.0428, *wR*_2_ = 0.1054 for *I* ≥ 2*σ* (*I*). The goodness of fit on *F*^2^ was 1.027. Flack parameter value was 0.02(13). CCDC number: 2113454.

Crystallographic data for **2**: C_30_H_30_O_11_·0.5H_2_O, *M*_r_ = 575.55, tetragonal, space group: *P*4_1_2_1_2, Z = 4, *a* = *b* = 7.53767 (3) Å, *c* = 46.8060 (2) Å, *α* = *β* = *γ* = 90°, *V* = 2659.35 (2) Å^3^, *D*_c_ = 1.438 g/cm^3^, *μ* = 0.935 mm^−1^, *T* = 150 K, *F*(000) = 1212.0. Crystal size: 0.28 × 0.15 × 0.06 mm^3^, 48,652 reflections collected (7.556° < 2*θ* < 143.304), and 2601 unique (*R*_int_ = 0.0355) were used in all calculations. The final *R*_1_ index was 0.0555, *wR*_2_ = 0.1723 for *I* ≥ 2*σ* (*I*). The goodness of fit on *F*^2^ was 1.125. Flack parameter value was −0.08 (5). CCDC number: 2113455.

### 3.5. Antimicrobial Assays

The compounds were dissolved in DMSO and antifungal and antibacterial activities were assayed in 96-well plates by a serial dilution test in the range of 0.1–100 μM for the tested compounds, according to the methods in the previously published article [39,40]. Ketoconazole and ampicillin were used as positive controls for antifungal and antibacterial tests, and DMSO was used as a blank control, respectively.

### 3.6. Computation Methods

Initial conformational analysis was conducted by Spartan’14 software using the Merck Molecular Force Field (MMFF) method [41]. The conformation with the Boltzmann population greater than 1% were optimized at the B3LYP/6–31 + G (d,p) level with the density functional theory (DFT) and then performed to TDDFT at the B3LYP/6–311 + G (d,p) level to afford calculated ECD spectra in acetonitrile [42]. The calculated ECD spectra were generated using the SpecDis program (University of Würzburg) with sigma = 0.3 eV [43]. A 1D potential energy scan was performed on the dihedral angle C8-C7-C7′-C8′ using the DFT method at the B3LYP/6-31g (d,p) level; the results were generated using GaussView 6 [44].

## 4. Conclusions

In this work, two asymmetric tetrahydroxanthone dimers with a 7,7′-linkage were described for the first time, reporting an unprecedented dimerization site of xanthones. The structures of the two new compounds were unambiguously identified by a range of methods, such as HRESIMS, NMR spectra, ECD calculations, and X-ray diffraction. Due to the rotation of the two units, part NMR signals in **2**, missed at room temperature but obtained at a low temperature of 243 K, and 1D PES scans, were performed to verify the existence of axial chirality. Concurrently, it also represented an axial chirality with rapid rotation in non-biaryl compounds. The new compounds exhibited broad-spectrum and potential antifungal and antibacterial activities against *C. gloeosporioides*, *F. oxysporum*, *F. oxysporum*, *C. musae*, and *C. albicans* with MIC values in the range of 3.13–25 μM. Additionally, compound **1** possessed moderate antibacterial activities against MRSA and *B. subtilis*. This work increases the diversity of connection modes of xanthone dimers, which is valuable for the chemical diversity of xanthone dimers, it also provides more evidence to support mangrove endophytic fungi as a sustainable source of chemical diversity.

## Figures and Tables

**Table 1:** ^1^H (500 MHz) and ^13^C (125 MHz) NMR data of compounds **1** and **2** in MeOD-*d*_4_ and CDCl_3_ *^a^*, *δ* in ppm.

	1		2 at 298 K		2 at 243 K		
Atom No.	*δ*_C_, Type	*δ*_H_ (Mult, *J* in Hz)	*δ*_C_, Type	*δ*_H_, (Mult, *J* in Hz)	*δ*_C_, Type		*δ*_H_, (Mult, *J* in Hz)
1	162.2, C		162.3, C		162.3, C	162.2, C	
2	107.6, CH	6.45, s	107.6, CH	6.45, s	107.2, CH	107.1, CH	6.45, s
3	153.6, C		153.8, C		153.7, C	153.3, C	
4	106.6, CH	6.42, s	106.7, CH	6.42, s	106.9, CH	106.9, CH	6.42, s
4a	159.3, C		159.2, C		159.3, C	159.2, C	
10a	81.5, C		81.4, C		81.5, C	81.4, C	
5	70.8, CH	4.20, dd (12.4, 4.2)	70.9, CH	4.10–4.05, m	70.9, CH		4.08–4.04, m
6	29.9, CH_2_	2.10–2.02, m 1.71–1.62, m			33.7, CH_2_		2.56–2.48, m 2.23–2.14, m
7	39.9, CH	3.44–3.35, m			38.2, CH		3.02–2.95, m
8	175.0, C				175.4, C	174.9, C	
8a	105.8, C				105.8, C		
9	188.4, C		188.4, C		188.3, C		
9a	105.3, C		105.2, C		105.8, C		
11	64.1, CH_2_	4.51, s	64.1, CH_2_	4.51, s	64.8, CH_2_		4.53, s
12	26.1, CH_3_	1.48, s	26.1, CH_3_	1.50, s	25.5, CH_3_		1.52, s
1′	162.4, C		162.5, C		162.1, C	161.9, C	
2′	110.7, CH	6.27, s	110.8, CH	6.25, s	110.7, CH	110.5, CH	6.25, s
3′	150.8, C		150.6, C		150.7, C	150.3, C	
4′	109.8, CH	6.26, s	106.7, CH	6.27, s	109.9, CH	109.9, CH	6.27, s
4a’	159.3, C		159.4, C		159.0, C	158.9, C	
10a’	81.8, C		81.5, C		81.2, C	81.0, C	
5′	71.8, CH	4.12, dd (4.3, 1.9)	71.9, CH	4.10–4.05, m	70.2, CH		4.13–4.09, m
6′	28.0, CH_2_	2.20–2.14, m 1.87–1.79, m			25.8, CH_2_		2.14–2.07, m 1.59–1.67, m
7′	37.1, CH	3.44–3.35, m			35.0, CH		3.80–3.73, m
8′	176.9, C				177.4, C	176.9, C	
8a’	107.8, C				107.8, C		
9′	188.6, C		188.4, C		188.3, C		
9a’	106.1, C		106.1, C		106.4, C		
11′	22.5, CH_3_	2.25, s	22.5, CH_3_	2.25, s	22.6, CH_3_		2.25, s
12′	20.1, CH_3_	1.45, s	20.1, CH_3_	1.50, s	27.0, CH_3_		1.45, s

*^a.^* The volume ratio of MeOD-*d*_4_ and CDCl_3_ was 1:1.

**Table 2:** Antifungal and antibacterial activities of **1** and **2**.

	MIC of Compounds/μM			
	1	2	Ketoconazole *^a^*	Ampicillin *^b^*
*F. oxysporum*	12.5	12.5	6.25	NT
*P. italicum*	50	>100	1.56	NT
*C. musae*	25	12.5	1.56	NT
*C. gloeosporioides*	3.13	>100	0.1	NT
*C. albicans*	12.5	25	0.1	NT
MRSA	12.5	>100	NT	0.39
*P. aeruginosa*	>100	>100	NT	0.19
*B. subtilis*	25	25	NT	0.39

*^a^* Positive control toward fungi. *^b^* Positive control toward bacteria.

## Data Availability

All data generated or analyzed in this study are available within the manuscript and are available from the corresponding authors upon request.

## Supplementary Materials

The following supporting information can be downloaded at https://www.mdpi.com/article/10.3390/molecules27092691/s1, including HRESIMS, 1D and 2D NMR spectra, Chiral HPLC separation profile, and cartesian coordinates for the low-energy optimized conformer of **1** and **2**.

## Sample Availability

Samples of the compounds are not available from the authors.
