# Supplementary material for: Two Antimicrobial Heterodimeric Tetrahydroxanthones with a 7,7′-Linkage from Mangrove Endophytic Fungus Aspergillus flavus QQYZ
Source: Molecules. 2022 Apr 22;27(9):2691. doi: 10.3390/molecules27092691 (PMC9103106; doi:10.3390/molecules27092691)
Supplement: Supplementary file 1 [file molecules-27-02691-s001.zip › molecules-1660129-supplementary.pdf]

Supplementary Information

Two Antimicrobial Heterodimeric Tetrahydroxanthones with a 7,7′-Linkage from Mangrove Endophytic Fungus *Aspergillus flavus* QQYZ

Zhenming Zang [1], Wencong Yang [1], Hui Cui [2], Runlin Cai [3], Chunyuan Li [4], Ge Zou [1], Bo Wang [1,*] and Zhigang She [1,*]

[1] School of Chemistry, Sun Yat-sen University, Guangzhou 510275, China; zangzhm@mail2.sysu.edu.cn (Z.Z.); yangwc6@mail2.sysu.edu.cn (W.Y.); zoug5@mail2.sysu.edu.cn (G.Z.)
[2] School of Pharmaceutical Sciences, Guangzhou University of Chinese Medicine, Guangzhou 510006, China; cuihui@gzucm.edu.cn
[3] College of Science, Shantou University, Shantou 515063, China; rlcai@stu.edu.cn
[4] College of Materials and Energy, South China Agricultural University, Guangzhou 510642, China; chunyuanli@scau.edu.cn
* Correspondence: ceswb@mail.sysu.edu.cn (B.W.); cesshzhg@mail.sysu.edu.cn (Z.S.); Tel.: +86-20-84113356 (Z.S.)

List of Supplementary Information

Content

Figure S1. HRESIMS of aflaxanthone A (**1**) 4

Figure S2. ^{1}H NMR spectrum of aflaxanthone A (**1**) in MeOD-d_4 and $CDCl_3$ at 500 MHz 4

Figure S3. ^{13}C NMR spectrum of aflaxanthone A (**1**) in MeOD-d_4 and $CDCl_3$ at 125 MHz5

Figure S4. HSQC spectrum of aflaxanthone A (**1**) in MeOD-d_4 and $CDCl_3$ at 500 MHz and 125 MHz 5

Figure S5. ^{1}H-^{1}H COSY spectrum of aflaxanthone A (**1**) in MeOD-d_4 and $CDCl_3$ at 500 MHz 6

Figure S6. HMBC spectrum of aflaxanthone A (**1**) in MeOD-d_4 and $CDCl_3$ at 500 MHz and 125 MHz 6

Figure S7. NOESY spectrum of aflaxanthone A (**1**) in MeOD-d_4 and $CDCl_3$ at 500 MHz . 7

Figure S8. HRESIMS of aflaxanthone B (**2**) 7

Figure S9. ^{1}H NMR spectrum of aflaxanthone B (**2**) in MeOD-d_4 and $CDCl_3$ at 500 MHz, 298K 8

Figure S10. ^{13}C NMR spectrum of aflaxanthone B (**2**) in MeOD-d_4 and $CDCl_3$ at 125 MHz, 298K 8

Figure S11. HSQC spectrum of aflaxanthone B (**2**) in MeOD-d_4 and $CDCl_3$ at 500 MHz and 125MHz, 298K 9

Figure S12. ^{1}H NMR spectrum of aflaxanthone B **(2**) in MeOD-d_4 and $CDCl_3$ at 500 MHz, 273K 9

Figure S13. ^{1}H NMR spectrum of aflaxanthone B (**2**) in MeOD-d_4 and $CDCl_3$ at 500 MHz, 243K 10

Figure S14. ^{13}C NMR spectrum of aflaxanthone B (**2**) in MeOD-d_4 and $CDCl_3$ at 125 MHz, 243K 10

Figure S15. HSQC spectrum of aflaxanthone B (**2**) in MeOD-d_4 and $CDCl_3$ at 500MHz and 125 MHz, 243K 11

Figure S16. ^{1}H-^{1}H COSY spectrum of aflaxanthone B (**2**) in MeOD-d_4 and $CDCl_3$ at 500 MHz, 243K 11

Figure S17. HMBC spectrum of aflaxanthone B (**2**) in MeOD-d_4 and $CDCl_3$ at 500MHz and 125 MHz, 243K 12

Figure S18. NOESY spectrum of aflaxanthone B (**2**) in MeOD-d_4 and $CDCl_3$ at 500MHz and 125 MHz, 243K 13

Figure S19. Chiral HPLC separation profile of **1** and **2**. 13

Table S1. Gibbs free energy and Boltzmann population of low energy of 5*S*,7*R*,10a*R*,5'*S*,7'*S*,10a'*S*-**1** in CH_3CN 14

Table S2. Cartesian coordinates for the low-energy optimized conformer of **1**a at B3LYP/6–311+g (d,p) level of theory in CH_3CN 14

Table S3. Cartesian coordinates for the low-energy optimized conformer of **1**b at B3LYP/6–311+g (d,p) level of theory in CH_3CN 16

Table S4. Cartesian coordinates for the low-energy optimized conformer of **1**c at B3LYP/6–311+g (d,p) level of theory in CH_3CN 17

Table S5. Cartesian coordinates for the low-energy optimized conformer of **1**d at B3LYP/6–311+g (d,p) level of theory in CH_3CN 19

Table S6. Cartesian coordinates for the low-energy optimized conformer of **1**e at B3LYP/6–311+g (d,p) level of theory in CH_3CN 21

Table S7. Cartesian coordinates for the low-energy optimized conformer of **1**f at B3LYP/6–311+g (d,p) level of theory in CH_3CN 23

Table S8. Cartesian coordinates for the low-energy optimized conformer of **1**g at B3LYP/6–311+g (d,p) level of theory in CH_3CN 25

Table S9. Cartesian coordinates for the low-energy optimized conformer of **1**h at B3LYP/6–311+g (d,p) level of theory in CH_3CN 26

Table S10. Cartesian coordinates for the low-energy optimized conformer of **1**i at B3LYP/6–311+g (d,p) level of theory in CH_3CN 28

Table S11. Cartesian coordinates for the low-energy optimized conformer of **1**j at B3LYP/6–311+g (d,p) level of theory in CH_3CN 30

Table S12. Gibbs free energy and Boltzmann population of low energy of 5*S*,7*R*,10a*R*,5'*S*,7'*R*,10a'*R*-**2** in CH_3CN 32

Table S13. Cartesian coordinates for the low-energy optimized conformer of **2**a at B3LYP/6–311+g (d,p) level of theory in CH_3CN 32

Table S14. Cartesian coordinates for the low-energy optimized conformer of **2**b at B3LYP/6–311+g (d,p) level of theory in CH_3CN 34

Table S15. Cartesian coordinates for the low-energy optimized conformer of **2**c at B3LYP/6–311+g (d,p) level of theory in CH_3CN 35

Table S16. Cartesian coordinates for the low-energy optimized conformer of **2**d at B3LYP/6–311+g (d,p) level of theory in CH_3CN 37

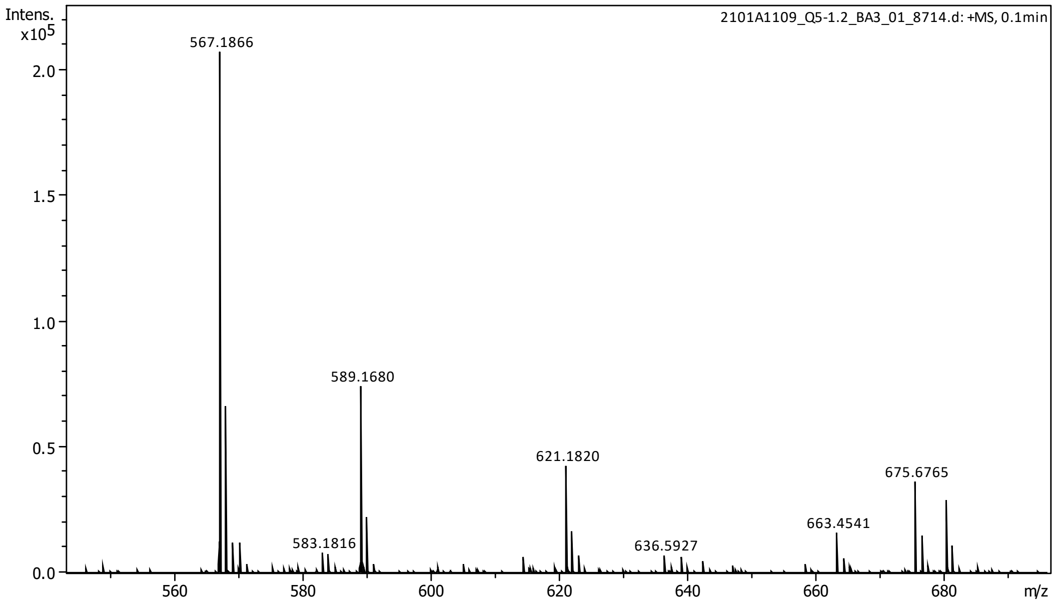

Figure S1. HRESIMS of aflaxanthone A (**1**).

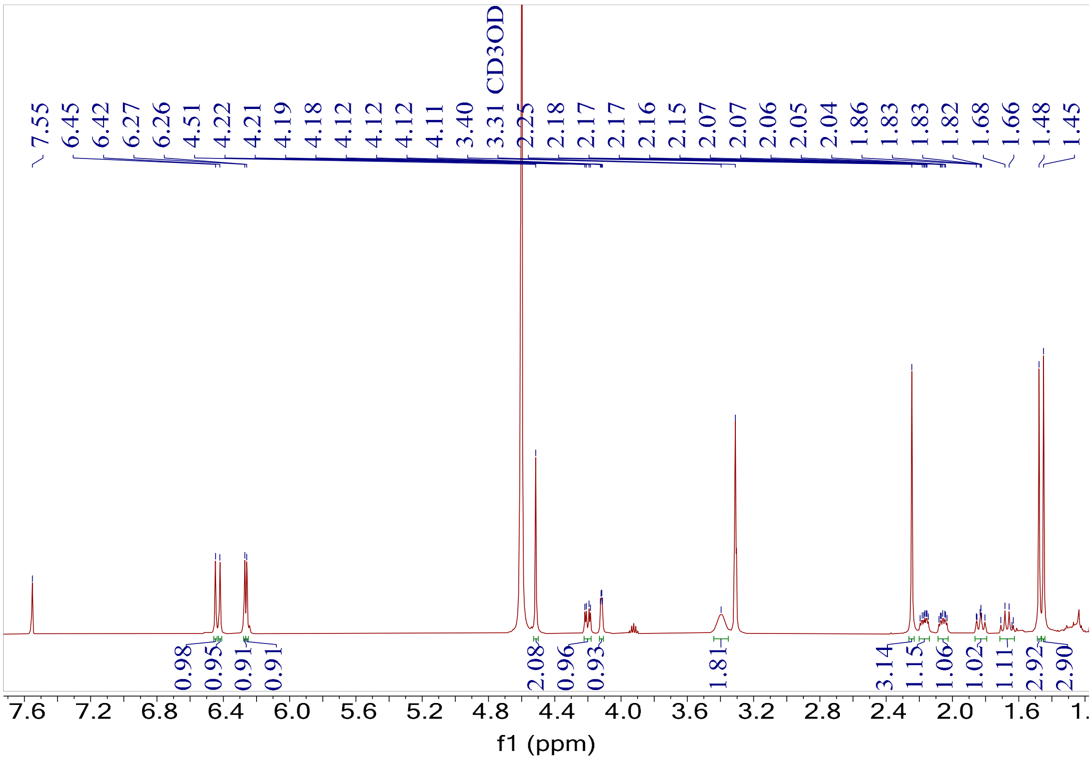

Figure S2. ^{1}H NMR spectrum of aflaxanthone A (**1**) in MeOD-d_4 and $CDCl_3$ at 500 MHz.

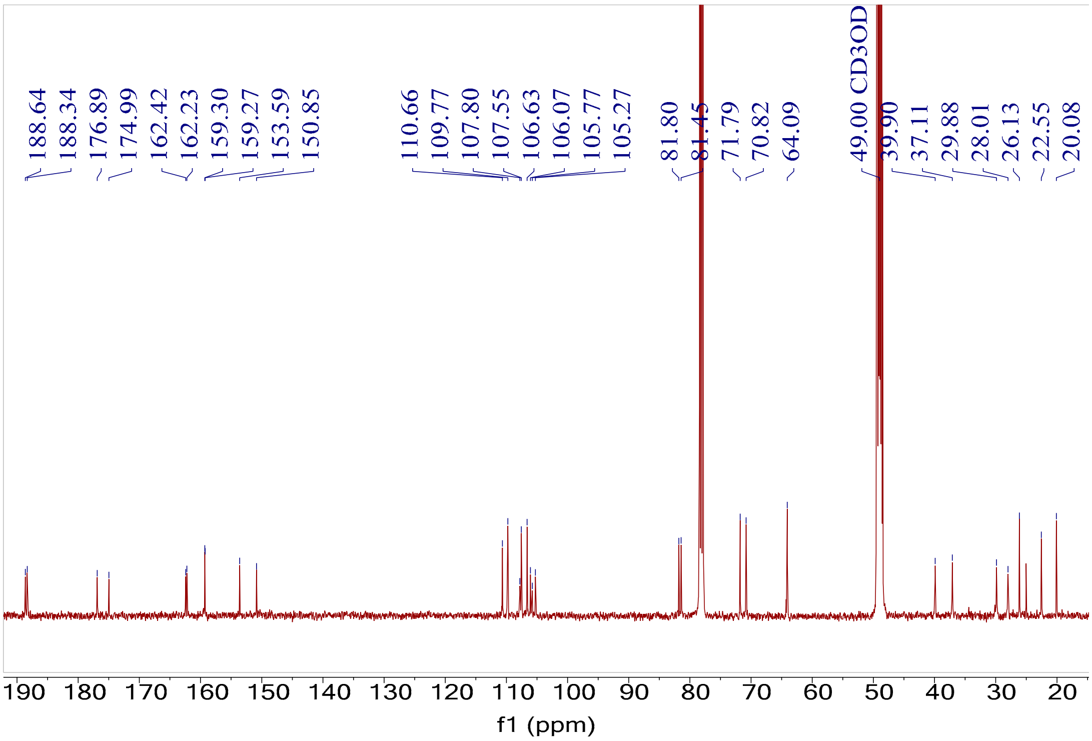

Figure S3. ^{13}C NMR spectrum of aflaxanthone A (**1**) in MeOD-d_4 and $CDCl_3$ at 125 MHz.

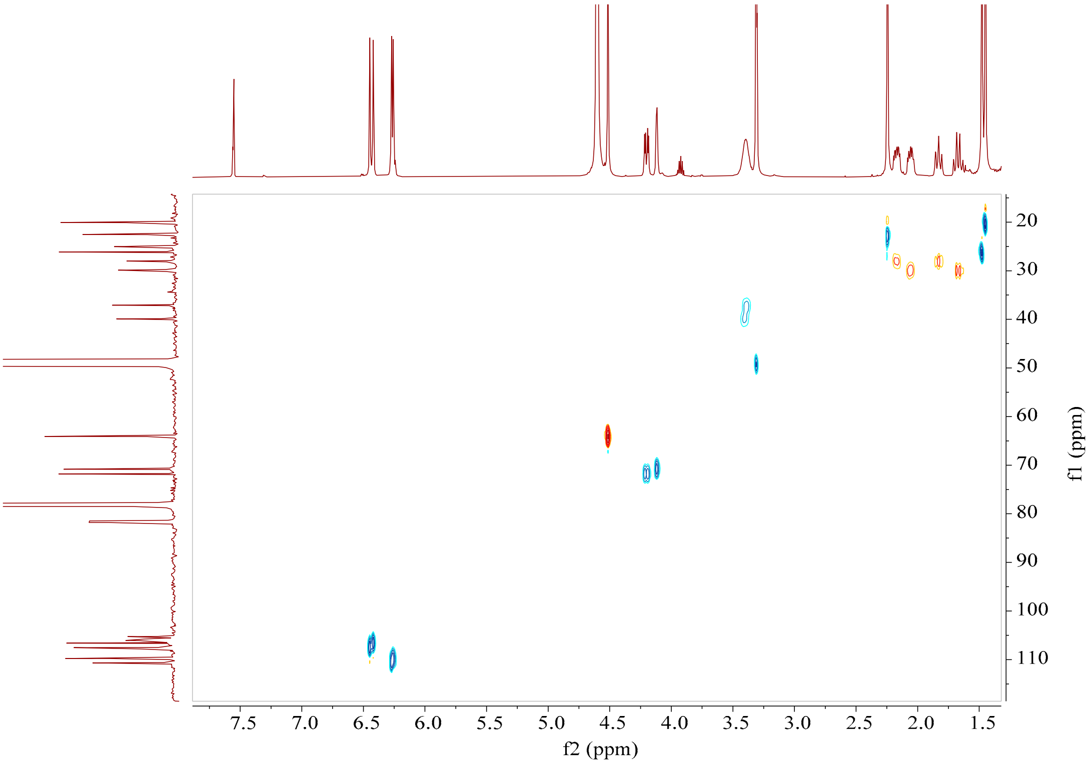

Figure S4. HSQC spectrum of aflaxanthone A (**1**) in MeOD-d_4 and $CDCl_3$ at 500 MHz and 125 MHz.

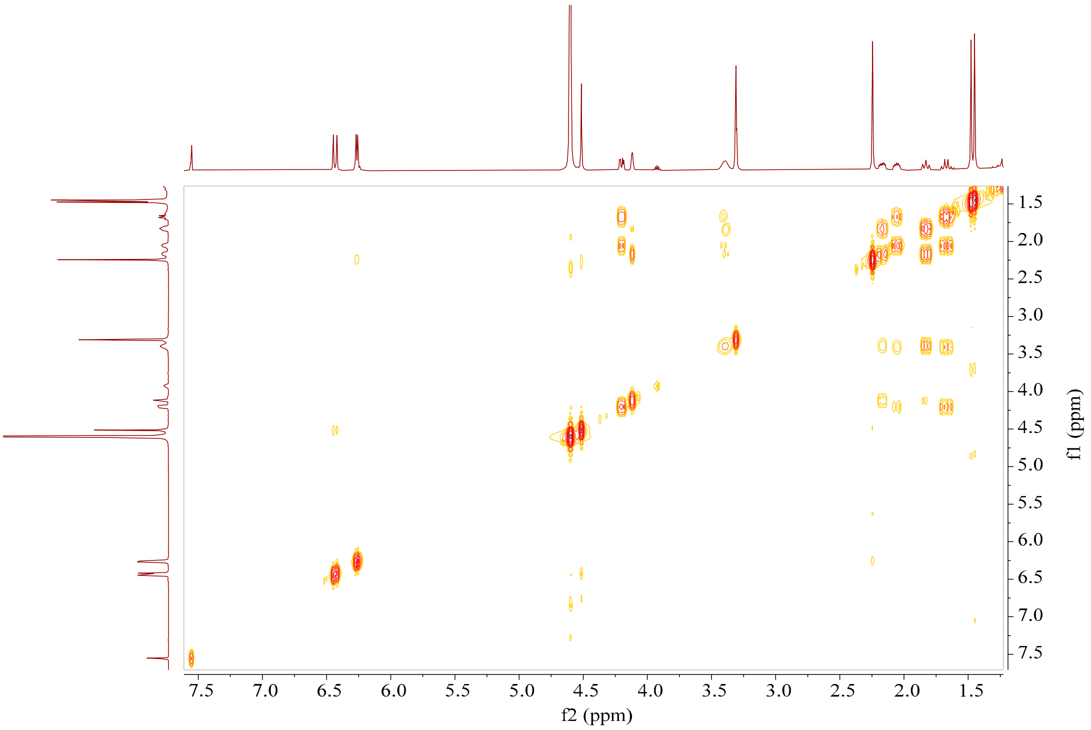

Figure S5. ^{1}H-^{1}H COSY spectrum of aflaxanthone A (**1**) in MeOD-d_4 and $CDCl_3$ at 500 MHz.

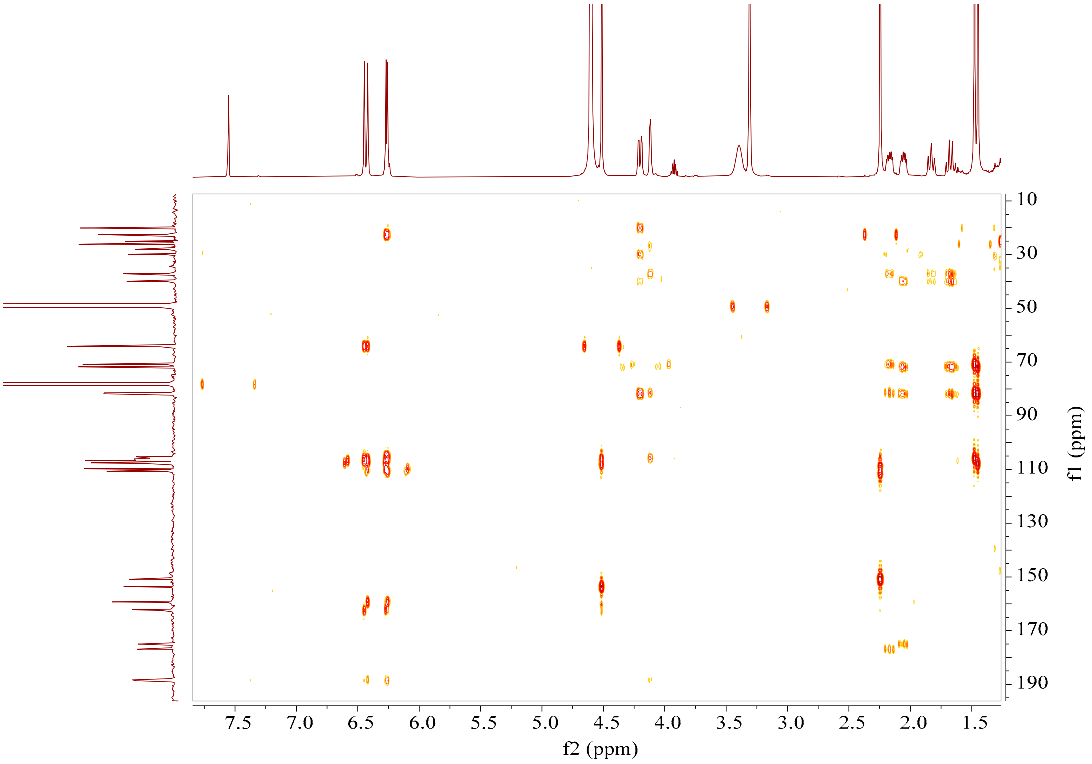

Figure S6. HMBC spectrum of aflaxanthone A (**1**) in MeOD-d_4 and $CDCl_3$ at 500 MHz and 125 MHz.

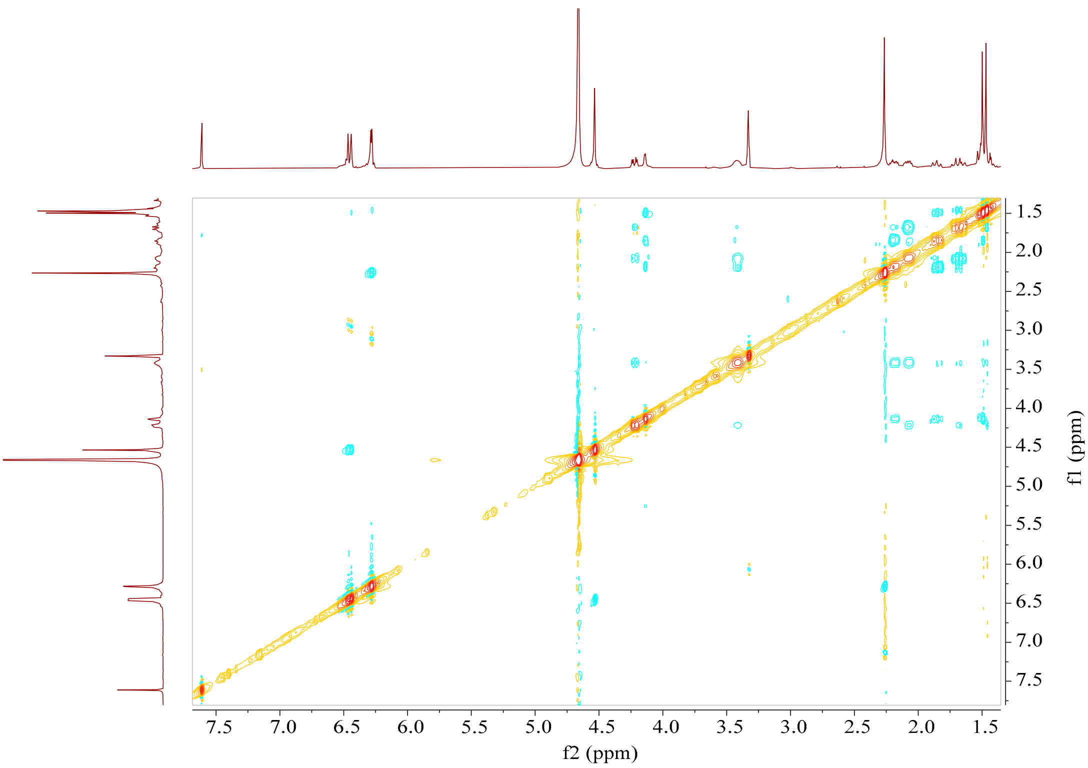

Figure S7. NOESY spectrum of aflaxanthone A (**1**) in MeOD-d_4 and $CDCl_3$ at 500 MHz.

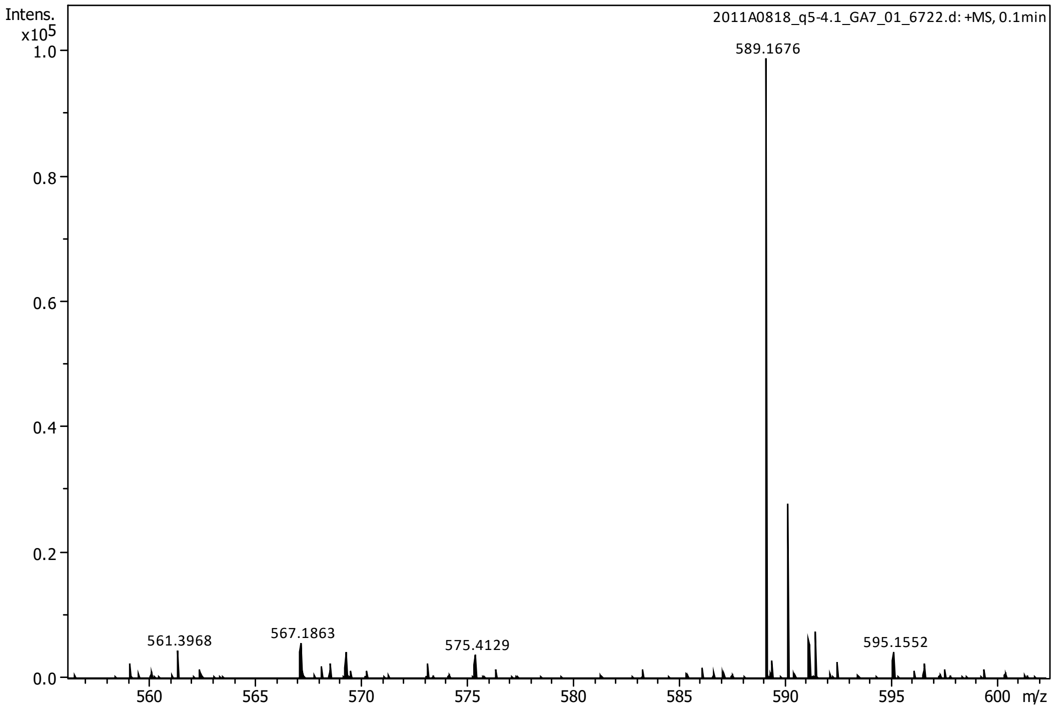

Figure S8. HRESIMS of aflaxanthone B (**2**).

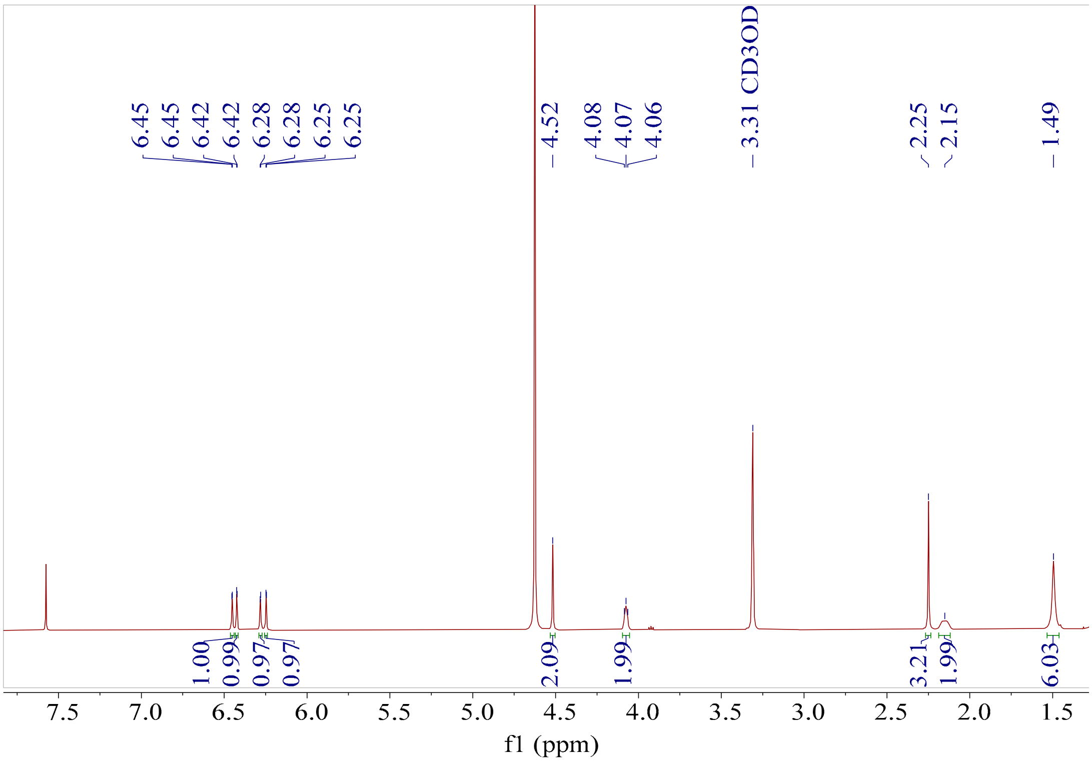

Figure S9. ^{1}H NMR spectrum of aflaxanthone B (**2**) in MeOD-d_4 and $CDCl_3$ at 500 MHz, 298K.

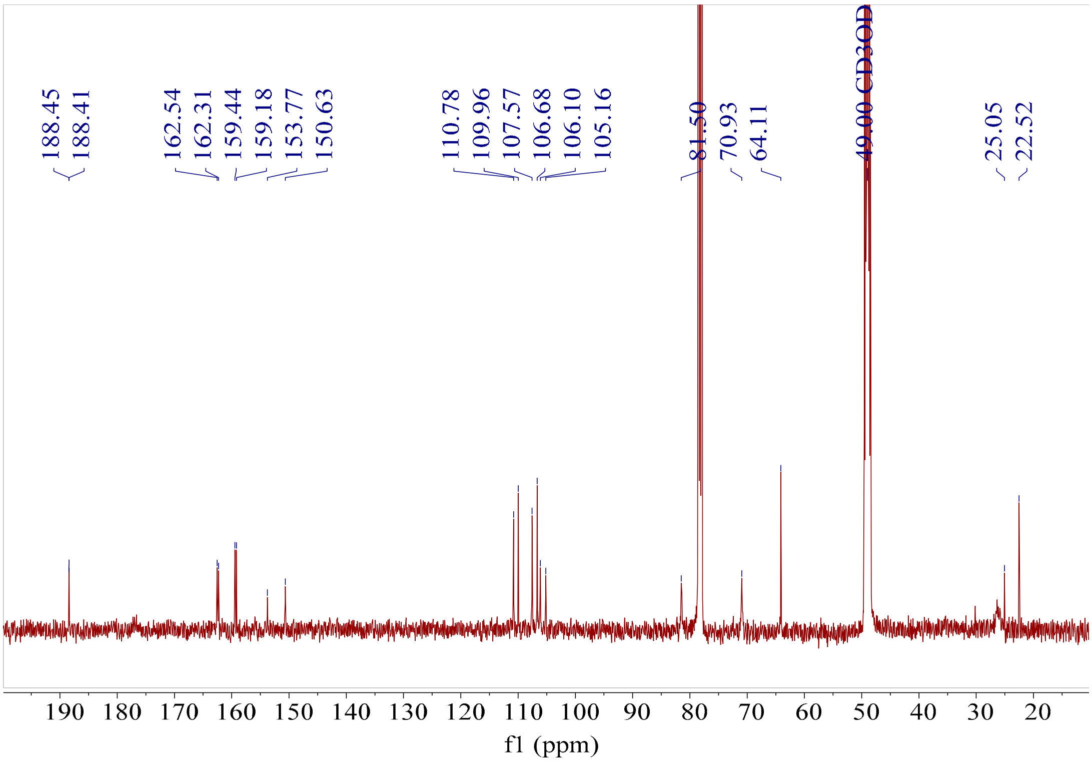

Figure S10. ^{13}C NMR spectrum of aflaxanthone B (**2**) in MeOD-d_4 and $CDCl_3$ at 125 MHz, 298K.

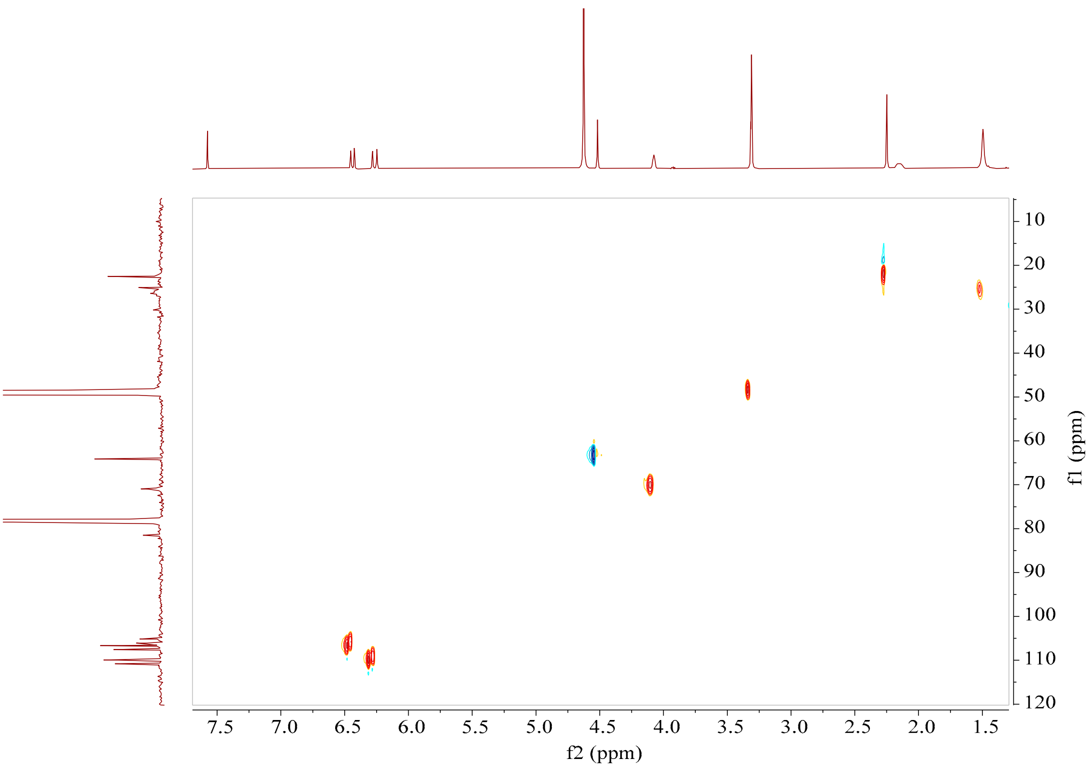

Figure S11. HSQC spectrum of aflaxanthone B (**2**) in MeOD-d_4 and $CDCl_3$ at 500 MHz and 125MHz, 298K.

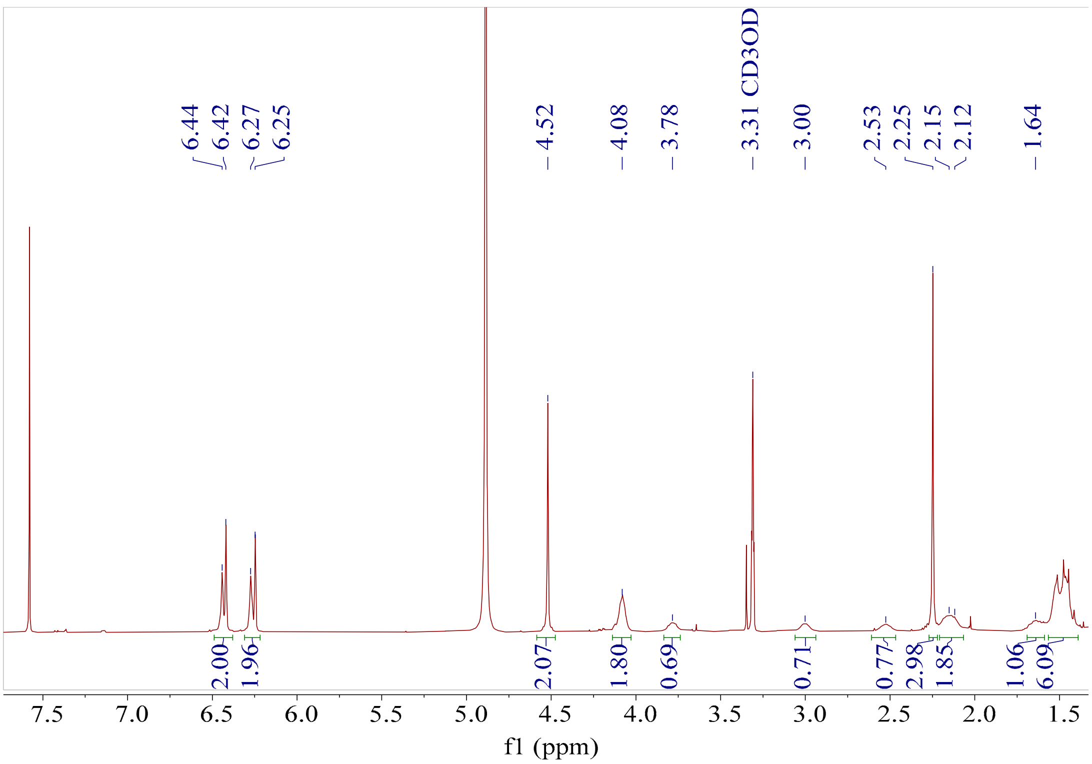

Figure S12. ^{1}H NMR spectrum of aflaxanthone B (**2**) in MeOD-d_4 and $CDCl_3$ at 500 MHz, 273K.

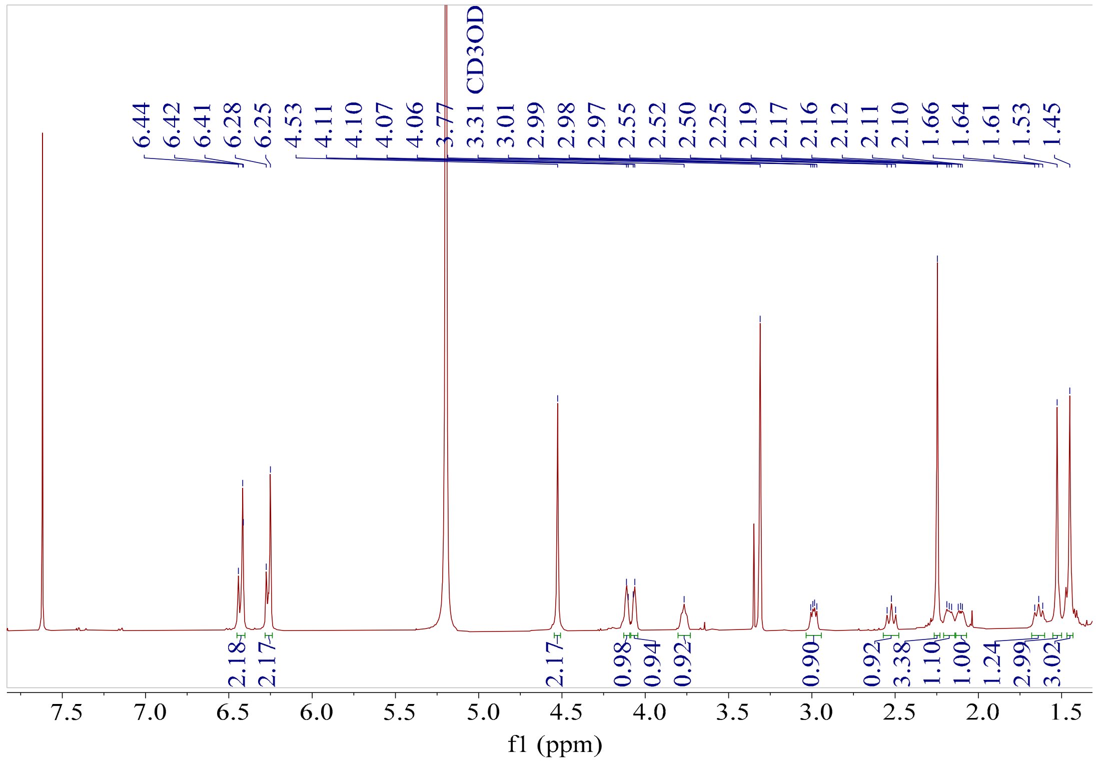

Figure S13. ^{1}H NMR spectrum of aflaxanthone B (**2**) in MeOD-d_4 and $CDCl_3$ at 500 MHz, 243K.

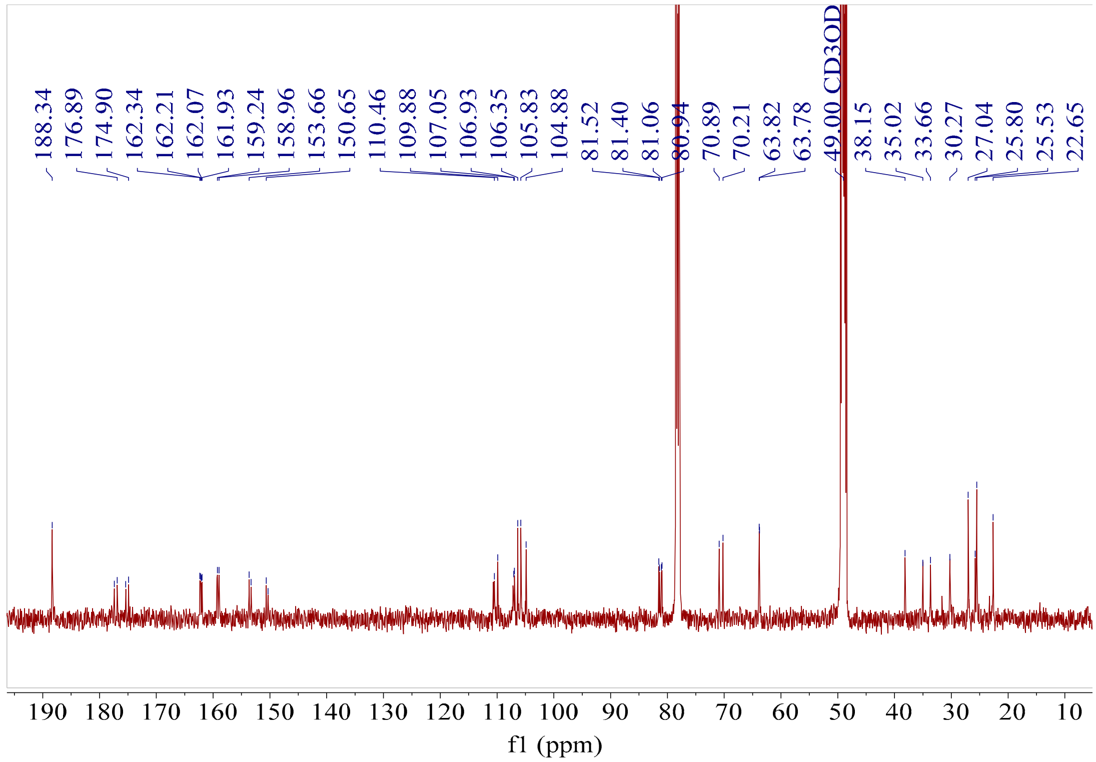

Figure S14. ^{13}C NMR spectrum of aflaxanthone B (**2**) in MeOD-d_4 and $CDCl_3$ at 125 MHz, 243K.

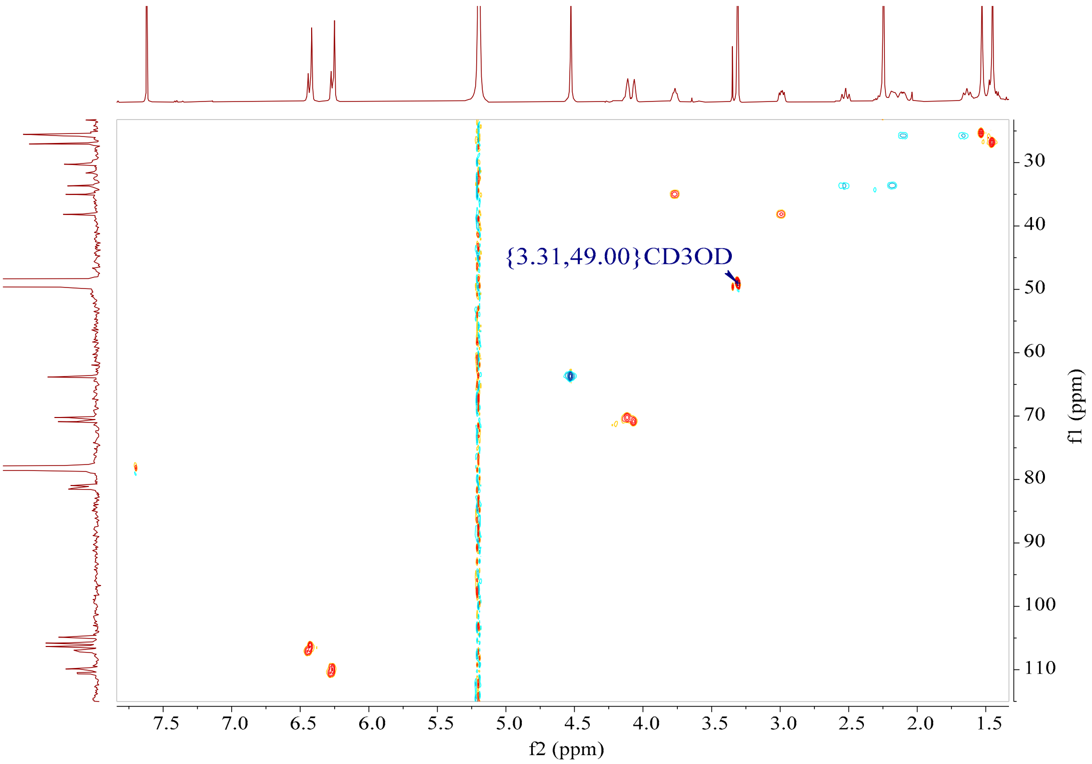

Figure S15. HSQC spectrum of aflaxanthone B (**2**) in MeOD-d_4 and $CDCl_3$ at 500MHz and 125 MHz, 243K.

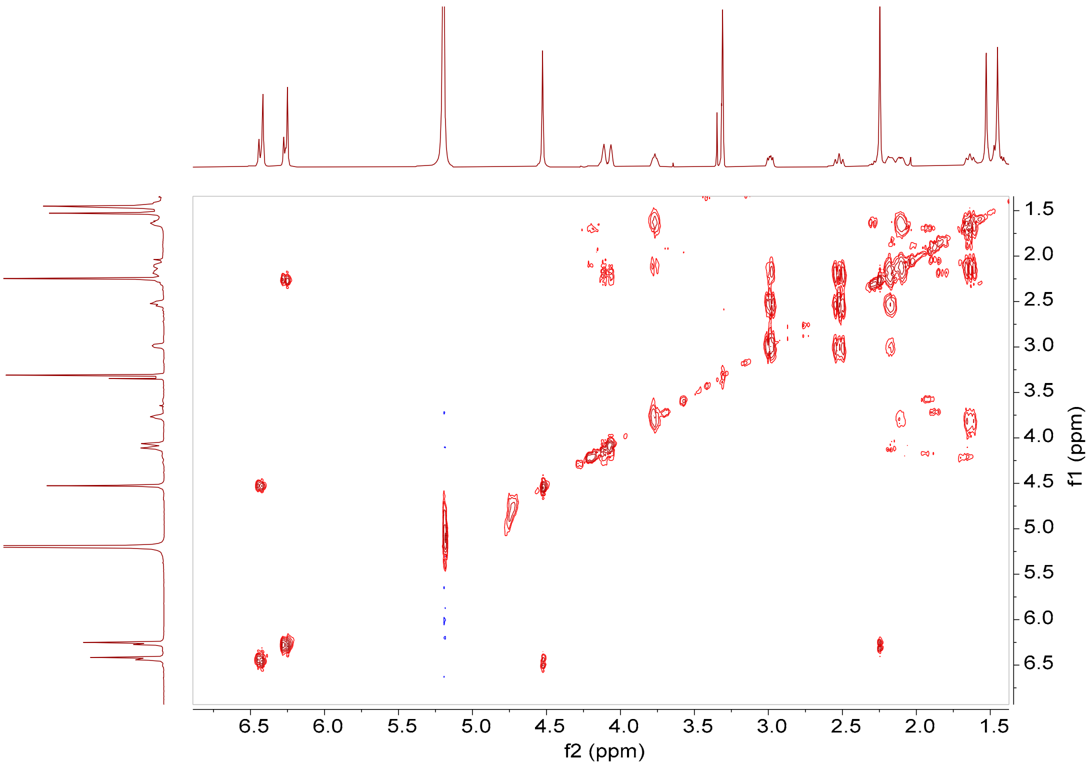

Figure S16. ^1H-^1H COSY spectrum of aflaxanthone B (**2**) in MeOD-d_4 and $CDCl_3$ at 500 MHz, 243K.

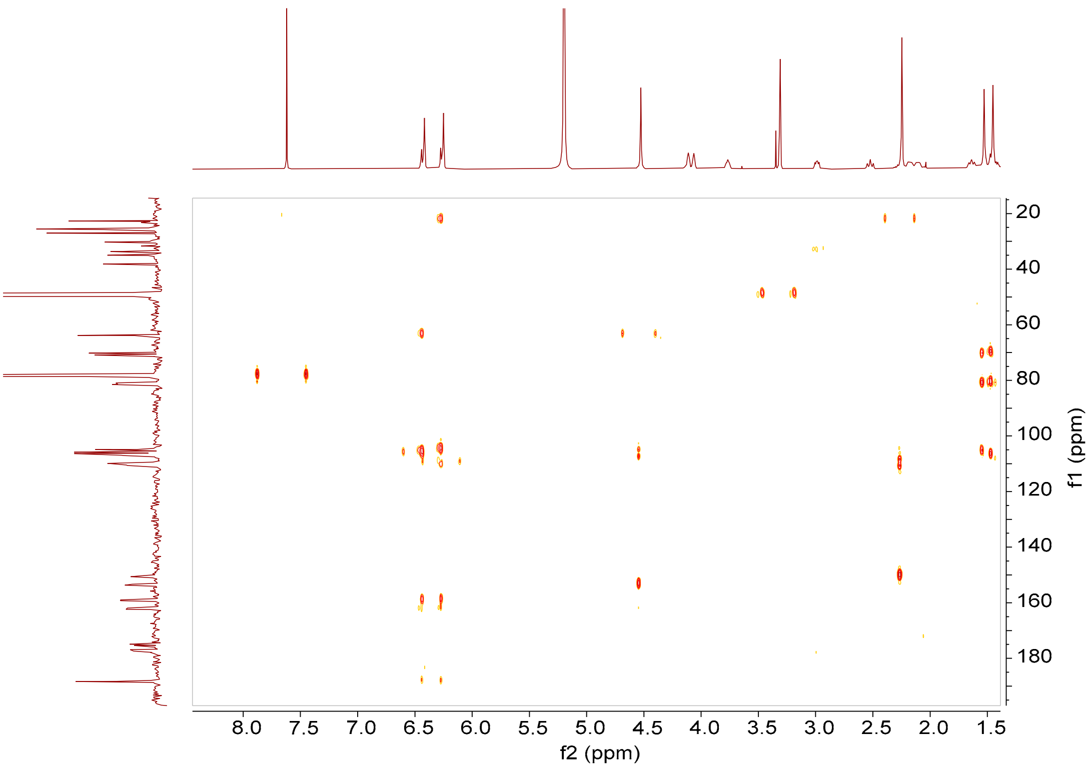

Figure S17. HMBC spectrum of aflaxanthone B (**2**) in MeOD-d_4 and $CDCl_3$ at 500MHz and 125 MHz, 243K.

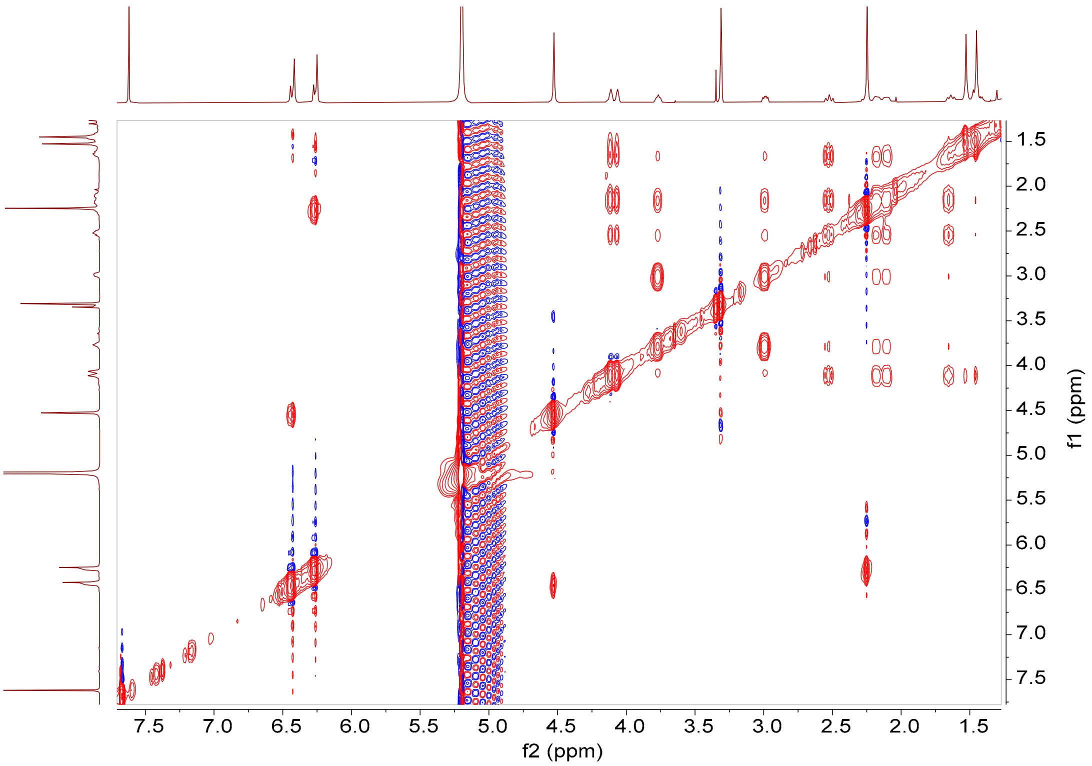

Figure S18. NOESY spectrum of aflaxanthone B (**2**) in MeOD-d_4 and $CDCl_3$ at 500MHz and 125 MHz, 243K.

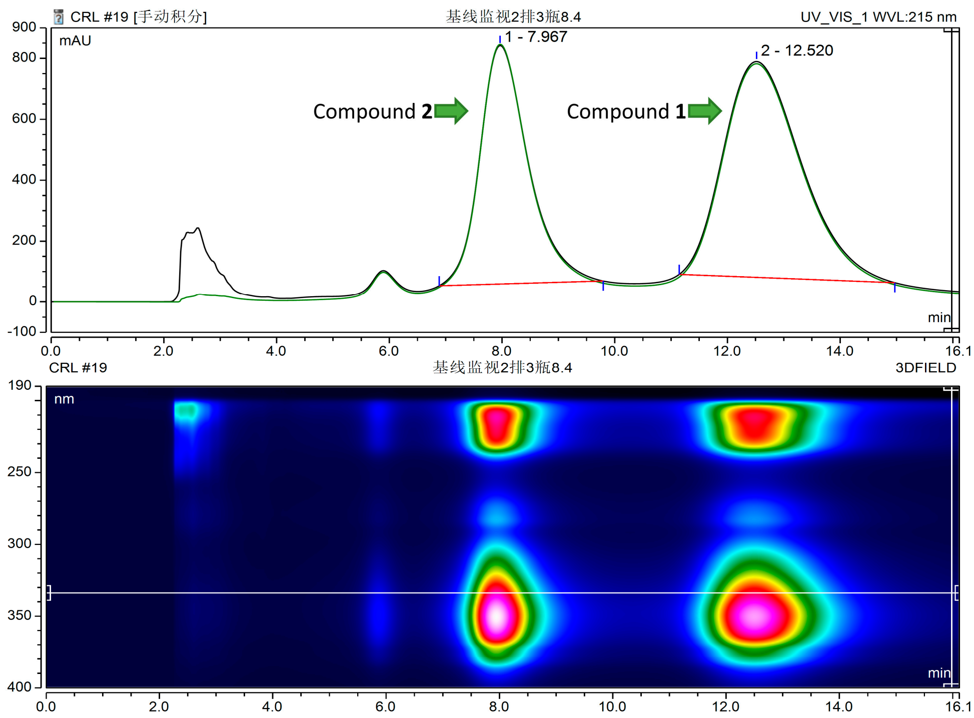

Figure S19. Chiral HPLC separation profile of **1** and **2**.

Table S1. Gibbs free energy and Boltzmann population of low energy of 5*S*,7*R*,10a*R*,5'*S*,7'*S*,10a'*S*-**1** in CH_3CN.

Conformers of 5*S*,7*R*,10a*R*,5'*S*,7'*S*,10a'*S*-1	Δ*G* (kcal/mol)	P
**1**a	0	0.266
**1**b	0.52	0.216
**1**c	0.62	0.207
**1**d	2.32	0.104
**1**e	3.38	0.068
**1**f	3.50	0.065
**1**g	6.44	0.020
**1**h	6.96	0.016
**1**i	7.01	0.016
**1**j	7.12	0.015

Table S2. Cartesian coordinates for the low-energy optimized conformer of **1**a at B3LYP/6–311+g (d,p) level of theory in CH_3CN.

**Conformer of 1a**			**Standard Orientation (Å)**		
Number	Atom	Type	X	Y	Z
1	6	0	6.836900	−2.763597	−0.219150
2	6	0	7.655909	−1.645137	−0.019158
3	6	0	7.062497	−0.389368	0.166795
4	6	0	5.673525	−0.247430	0.117251
5	6	0	4.864602	−1.363147	−0.080341
6	6	0	5.450526	−2.622572	−0.246482
7	8	0	5.191647	1.018512	0.300799
8	6	0	3.873174	1.282584	−0.235470
9	6	0	2.888530	0.184734	0.078742
10	6	0	3.405454	−1.198271	−0.054110
11	6	0	3.423812	2.602042	0.458651
12	6	0	1.955429	2.910965	0.183189
13	6	0	1.030577	1.791545	0.704982
14	6	0	1.617877	0.398039	0.481716
15	8	0	4.724796	−3.769330	−0.431533
16	6	0	9.146127	−1.801349	0.057487
17	8	0	2.659419	−2.173443	−0.121602
18	8	0	0.742061	−0.637694	0.763020
19	6	0	4.004765	1.474184	−1.762587
20	1	0	3.580723	2.524269	1.543258
21	8	0	4.209582	3.717092	0.022991
22	6	0	−0.425197	2.011120	0.161962
23	1	0	1.008907	1.918103	1.797754
24	6	0	−0.545650	1.649067	−1.340289
25	6	0	−1.993415	1.458316	−1.779646
26	6	0	−2.729968	0.377992	−0.933379

27	6	0	−2.521889	0.639066	0.536468
28	6	0	−1.497921	1.362382	1.032230
29	8	0	−4.118440	0.488131	−1.330796
30	6	0	−5.027756	−0.119471	−0.512565
31	6	0	−4.809488	−0.361908	0.839512
32	6	0	−3.547430	0.077577	1.447077
33	6	0	−6.247179	−0.469391	−1.097081
34	6	0	−7.248448	−1.104490	−0.346433
35	6	0	−7.017779	−1.344000	1.016019
36	6	0	−5.809401	−0.972617	1.603248
37	8	0	−5.679896	−1.230290	2.941579
38	8	0	−3.375272	−0.011770	2.661690
39	8	0	−1.355120	1.633633	2.384753
40	8	0	−2.659960	2.728472	−1.691154
41	1	0	−2.022753	1.183606	−2.841659
42	6	0	−2.224838	−1.025766	−1.323391
43	6	0	−8.576577	−1.466048	−0.956666
44	8	0	−8.417078	−1.828784	−2.321647
45	1	0	−0.633735	3.089603	0.239846
46	1	0	7.272638	−3.752355	−0.344767
47	1	0	7.683563	0.486077	0.343844
48	1	0	1.687642	3.861390	0.663541
49	1	0	1.811634	3.088304	−0.887831
50	1	0	3.770288	−3.543646	−0.371687
51	1	0	9.485098	−2.628206	−0.576159
52	1	0	9.652222	−0.896189	−0.293003
53	1	0	9.448125	−1.999801	1.090321
54	1	0	1.180415	−1.494395	0.564642
55	1	0	4.753681	2.237880	−2.001784
56	1	0	4.346601	0.558078	−2.258336
57	1	0	3.056228	1.758473	−2.228199
58	1	0	5.136473	3.505217	0.243446
59	1	0	0.020620	0.743025	−1.578567
60	1	0	−0.098817	2.458302	−1.931295
61	1	0	−6.414081	−0.261441	−2.153066
62	1	0	−7.780733	−1.815059	1.631867
63	1	0	−4.813019	−0.873963	3.238993
64	1	0	−2.004366	1.088412	2.882514
65	1	0	−3.612036	2.546121	−1.803942
66	1	0	−2.767745	−1.814939	−0.791590
67	1	0	−2.389176	−1.210227	−2.391658
68	1	0	−1.160361	−1.161624	−1.112165
69	1	0	−9.256718	−0.611350	−0.893288

70	1	0	−9.020557	−2.320225	−0.433792
71	1	0	−9.295273	−2.089997	−2.643541

Table S3. Cartesian coordinates for the low-energy optimized conformer of **1**b at B3LYP/6–311+g (d,p) level of theory in CH_3CN.

**Conformer of 1b**			**Standard Orientation (Å)**		
Number	Atom	Type	X	Y	Z
1	6	0	6.912036	−2.656698	−0.386926
2	6	0	7.706833	−1.544110	−0.089158
3	6	0	7.087502	−0.316818	0.188486
4	6	0	5.697414	−0.196476	0.131170
5	6	0	4.911920	−1.308254	−0.162882
6	6	0	5.523119	−2.539433	−0.420936
7	8	0	5.188685	1.041100	0.408217
8	6	0	3.872207	1.323850	−0.122862
9	6	0	2.905431	0.186967	0.092497
10	6	0	3.449693	−1.172202	−0.143398
11	6	0	3.389796	2.575954	0.666203
12	6	0	1.919271	2.878761	0.398291
13	6	0	1.010143	1.705044	0.819954
14	6	0	1.625864	0.344002	0.494103
15	8	0	4.822034	−3.680694	−0.705663
16	6	0	9.199051	−1.678786	−0.006131
17	8	0	2.722962	−2.151687	−0.297167
18	8	0	0.766873	−0.725904	0.681223
19	6	0	4.017586	1.636914	−1.628406
20	1	0	3.536788	2.417104	1.743359
21	8	0	4.158331	3.736249	0.328442
22	6	0	−0.444259	1.939655	0.279197
23	1	0	0.975613	1.744988	1.918549
24	6	0	−0.542648	1.696386	−1.248100
25	6	0	−1.982807	1.512138	−1.717891
26	6	0	−2.704999	0.353469	−0.967925
27	6	0	−2.517793	0.502183	0.520707
28	6	0	−1.512346	1.203984	1.083588
29	8	0	−4.091335	0.464245	−1.373820
30	6	0	−4.999129	−0.214527	−0.611206
31	6	0	−4.789126	−0.557541	0.720278
32	6	0	−3.538864	−0.148255	1.373168
33	6	0	−6.208272	−0.532700	−1.232417
34	6	0	−7.209219	−1.226876	−0.535465
35	6	0	−6.986588	−1.579652	0.801518
36	6	0	−5.786773	−1.239780	1.425206

37	8	0	−5.662535	−1.602198	2.739344
38	8	0	−3.375403	−0.333834	2.578552
39	8	0	−1.388993	1.370644	2.453924
40	8	0	−2.674675	2.757270	−1.536236
41	1	0	−1.995326	1.321465	−2.797987
42	6	0	−2.165909	−1.004615	−1.459707
43	6	0	−8.506582	−1.566507	−1.216949
44	8	0	−9.236065	−0.381372	−1.499157
45	1	0	−0.674684	3.004697	0.439911
46	1	0	7.367485	−3.624766	−0.585276
47	1	0	7.690071	0.552225	0.441695
48	1	0	1.627466	3.784007	0.946938
49	1	0	1.782905	3.136309	−0.658082
50	1	0	3.863375	−3.478151	−0.639981
51	1	0	9.560913	−2.446128	−0.697257
52	1	0	9.691466	−0.738948	−0.276831
53	1	0	9.493352	−1.952149	1.012452
54	1	0	1.225703	−1.555507	0.424315
55	1	0	4.754322	2.429941	−1.797895
56	1	0	4.382146	0.768746	−2.189931
57	1	0	3.069132	1.939828	−2.083028
58	1	0	5.085794	3.525588	0.544219
59	1	0	0.044237	0.823208	−1.551829
60	1	0	−0.106673	2.558094	−1.767942
61	1	0	−6.374556	−0.243108	−2.269581
62	1	0	−7.746719	−2.110098	1.370684
63	1	0	−4.803930	−1.257958	3.074142
64	1	0	−2.028295	0.774274	2.900995
65	1	0	−3.621402	2.570857	−1.683993
66	1	0	−2.699632	−1.844141	−0.998443
67	1	0	−2.315727	−1.107865	−2.540490
68	1	0	−1.102167	−1.136112	−1.245774
69	1	0	−9.130806	−2.210066	−0.588760
70	1	0	−8.318833	−2.099056	−2.154721
71	1	0	−9.066359	0.245689	−0.774762

Table S4. Cartesian coordinates for the low-energy optimized conformer of **1**c at B3LYP/6–311+g (d,p) level of theory in CH_3CN.

**Conformer of 1c**			**Standard Orientation (Å)**		
Number	Atom	Type	X	Y	Z
1	6	0	6.810195	−2.794750	−0.176221
2	6	0	7.636233	−1.682886	0.025192
3	6	0	7.050819	−0.421907	0.201831

4	6	0	5.663856	−0.268979	0.140222
5	6	0	4.847960	−1.379274	−0.056756
6	6	0	5.425327	−2.644273	−0.213343
7	8	0	5.189315	1.000805	0.314298
8	6	0	3.878226	1.273021	−0.232045
9	6	0	2.882496	0.184496	0.080120
10	6	0	3.390254	−1.203426	−0.042918
11	6	0	3.432470	2.599236	0.451330
12	6	0	1.968598	2.917698	0.164333
13	6	0	1.033180	1.807284	0.686120
14	6	0	1.610406	0.408767	0.472888
15	8	0	4.693227	−3.785522	−0.399355
16	6	0	9.124717	−1.848949	0.113838
17	8	0	2.636696	−2.173291	−0.111922
18	8	0	0.725088	−0.618646	0.752197
19	6	0	4.022278	1.455979	−1.760235
20	1	0	3.582003	2.526966	1.536795
21	8	0	4.229595	3.707084	0.015161
22	6	0	−0.419225	2.035129	0.133216
23	1	0	1.005603	1.940449	1.777051
24	6	0	−0.531759	1.667817	−1.367832
25	6	0	−1.979701	1.483339	−1.815587
26	6	0	−2.727455	0.412492	−0.968286
27	6	0	−2.526839	0.679262	0.501477
28	6	0	−1.499889	1.398283	1.000596
29	8	0	−4.112281	0.524828	−1.376941
30	6	0	−5.033935	−0.060891	−0.557133
31	6	0	−4.824479	−0.298995	0.797861
32	6	0	−3.560684	0.128755	1.409841
33	6	0	−6.255517	−0.398634	−1.144600
34	6	0	−7.272013	−0.998474	−0.386780
35	6	0	−7.045643	−1.254701	0.972017
36	6	0	−5.834394	−0.897787	1.560328
37	8	0	−5.707615	−1.161748	2.896858
38	8	0	−3.393704	0.037674	2.624410
39	8	0	−1.364351	1.673908	2.351564
40	8	0	−2.636995	2.758617	−1.735993
41	1	0	−2.004549	1.204655	−2.875640
42	6	0	−2.227063	−0.996706	−1.347697
43	6	0	−8.574298	−1.378255	−1.037055
44	8	0	−8.376243	−2.474971	−1.917405
45	1	0	−0.619110	3.115378	0.205922
46	1	0	7.239728	−3.787520	−0.294125

47	1	0	7.677599	0.450772	0.378910
48	1	0	1.704652	3.872940	0.637246
49	1	0	1.833766	3.090261	−0.909041
50	1	0	3.739791	−3.553359	−0.346503
51	1	0	9.462104	−2.681525	−0.512920
52	1	0	9.639968	−0.948491	−0.236601
53	1	0	9.417948	−2.044708	1.149731
54	1	0	1.159732	−1.479165	0.563664
55	1	0	4.779011	2.212150	−1.997707
56	1	0	4.360391	0.534460	−2.248738
57	1	0	3.079211	1.744623	−2.234537
58	1	0	5.152629	3.489886	0.243253
59	1	0	0.028564	0.757612	−1.600070
60	1	0	−0.076792	2.471535	−1.959294
61	1	0	−6.414895	−0.202305	−2.204061
62	1	0	−7.814360	−1.722284	1.584943
63	1	0	−4.841649	−0.807937	3.200359
64	1	0	−2.017199	1.135141	2.849352
65	1	0	−3.589910	2.585015	−1.859478
66	1	0	−2.779951	−1.779064	−0.815837
67	1	0	−2.385730	−1.185256	−2.415656
68	1	0	−1.165851	−1.138467	−1.127226
69	1	0	−8.980120	−0.535132	−1.605752
70	1	0	−9.323177	−1.666387	−0.291674
71	1	0	−7.695988	−3.046159	−1.523676

Table S5. Cartesian coordinates for the low-energy optimized conformer of **1**d at B3LYP/6–311+g (d,p) level of theory in CH_3CN.

**Conformer of 1d**			**Standard Orientation (Å)**		
Number	Atom	Type	X	Y	Z
1	6	0	−7.402831	−1.581902	−0.819087
2	6	0	−7.586386	−1.438501	0.561329
3	6	0	−6.601578	−0.787504	1.315850
4	6	0	−5.433616	−0.321654	0.707600
5	6	0	−5.256912	−0.466503	−0.665402
6	6	0	−6.246944	−1.095282	−1.428235
7	8	0	−4.532369	0.297225	1.525651
8	6	0	−3.155033	0.301671	1.070654
9	6	0	−3.026775	0.661317	−0.386953
10	6	0	−4.055858	0.097510	−1.292418
11	6	0	−2.477627	1.410030	1.928005
12	6	0	−1.051359	1.699035	1.472113
13	6	0	−0.983483	2.115677	−0.013433

14	6	0	−2.068158	1.476936	−0.874305
15	8	0	−6.157214	−1.261527	−2.784626
16	6	0	−8.849969	−1.919073	1.211874
17	8	0	−3.934217	0.104375	−2.516172
18	8	0	−2.012824	1.856726	−2.204910
19	6	0	−2.556949	−1.088624	1.368206
20	1	0	−3.067321	2.334479	1.869305
21	8	0	−2.441345	1.056377	3.317086
22	6	0	0.463160	1.952691	−0.607045
23	1	0	−1.218493	3.191850	−0.040877
24	6	0	1.385751	3.054895	−0.031213
25	6	0	2.861765	2.780587	−0.307039
26	6	0	3.316220	1.412021	0.278819
27	6	0	2.342797	0.326745	−0.105983
28	6	0	1.065459	0.553352	−0.481904
29	8	0	4.641142	1.197743	−0.259519
30	6	0	5.134285	−0.071669	−0.155915
31	6	0	4.336199	−1.206265	−0.045718
32	6	0	2.875982	−1.055942	−0.071672
33	6	0	6.524824	−0.197905	−0.199357
34	6	0	7.135946	−1.456818	−0.092045
35	6	0	6.322967	−2.593451	0.020987
36	6	0	4.935011	−2.467805	0.040533
37	8	0	4.220828	−3.632530	0.137403
38	8	0	2.141927	−2.043633	−0.079708
39	8	0	0.197668	−0.470187	−0.823664
40	8	0	3.074958	2.832413	−1.725518
41	1	0	3.472163	3.589268	0.113679
42	6	0	3.423077	1.517946	1.814805
43	6	0	8.632500	−1.608057	−0.159481
44	8	0	9.272164	−0.500642	0.460752
45	1	0	0.405877	2.145168	−1.688499
46	1	0	−8.162508	−2.063513	−1.431749
47	1	0	−6.737995	−0.648588	2.386095
48	1	0	−0.634316	2.496016	2.102219
49	1	0	−0.422568	0.827005	1.681852
50	1	0	−5.330482	−0.826019	−3.092593
51	1	0	−9.249011	−2.796044	0.690145
52	1	0	−8.665518	−2.215694	2.250221
53	1	0	−9.603118	−1.125691	1.198539
54	1	0	−2.626501	1.288040	−2.720166
55	1	0	−2.728535	−1.375570	2.411510
56	1	0	−3.029857	−1.869728	0.760795

57	1	0	−1.482681	−1.131993	1.171429
58	1	0	−3.361525	0.853445	3.570374
59	1	0	1.237208	3.170664	1.047583
60	1	0	1.102245	4.011419	−0.488063
61	1	0	7.141443	0.693505	−0.301118
62	1	0	6.763299	−3.586401	0.086362
63	1	0	3.264574	−3.411842	0.086445
64	1	0	0.652635	−1.331338	−0.697436
65	1	0	3.974128	2.487558	−1.883483
66	1	0	3.831033	0.602965	2.259353
67	1	0	4.109997	2.323780	2.098231
68	1	0	2.453812	1.706803	2.288646
69	1	0	8.950295	−1.662523	−1.205587
70	1	0	8.953318	−2.515931	0.362936
71	1	0	10.227855	−0.672542	0.418210

Table S6. Cartesian coordinates for the low-energy optimized conformer of **1**e at B3LYP/6–311+g (d,p) level of theory in CH_3CN.

**Conformer of 1e**			**Standard Orientation (Å)**		
Number	Atom	Type	X	Y	Z
1	6	0	7.362176	−1.669221	0.851049
2	6	0	7.580768	−1.450893	−0.515154
3	6	0	6.619655	−0.750322	−1.255845
4	6	0	5.440008	−0.309944	−0.650167
5	6	0	5.228872	−0.529598	0.707513
6	6	0	6.195123	−1.208345	1.458220
7	8	0	4.563059	0.361928	−1.454378
8	6	0	3.175807	0.351325	−1.033211
9	6	0	3.013798	0.631146	0.438595
10	6	0	4.015748	0.007457	1.335972
11	6	0	2.527298	1.511889	−1.843522
12	6	0	1.091948	1.787705	−1.406345
13	6	0	0.991035	2.122095	0.097625
14	6	0	2.049529	1.425546	0.947630
15	8	0	6.069258	−1.450360	2.799407
16	6	0	8.857039	−1.904548	−1.160250
17	8	0	3.862899	−0.053488	2.554748
18	8	0	1.963041	1.730025	2.295706
19	6	0	2.575454	−1.014833	−1.421910
20	1	0	3.122124	2.426317	−1.719623
21	8	0	2.521535	1.236434	−3.249944
22	6	0	−0.469969	1.939842	0.647002
23	1	0	1.234145	3.192048	0.191293

24	6	0	−1.369788	3.081808	0.111100
25	6	0	−2.854680	2.807994	0.336470
26	6	0	−3.305751	1.479019	−0.335949
27	6	0	−2.351804	0.363999	0.010222
28	6	0	−1.081955	0.556203	0.427771
29	8	0	−4.647496	1.248238	0.153541
30	6	0	−5.147339	−0.010171	−0.020987
31	6	0	−4.355551	−1.145143	−0.175971
32	6	0	−2.895373	−1.009330	−0.114512
33	6	0	−6.539160	−0.126364	−0.008106
34	6	0	−7.154620	−1.376174	−0.180691
35	6	0	−6.351890	−2.511381	−0.349063
36	6	0	−4.962551	−2.395526	−0.340364
37	8	0	−4.256044	−3.557861	−0.490016
38	8	0	−2.170583	−2.003124	−0.150155
39	8	0	−0.231136	−0.493306	0.732432
40	8	0	−3.101442	2.781357	1.749333
41	1	0	−3.447317	3.644350	−0.053048
42	6	0	−3.372025	1.670502	−1.867030
43	6	0	−8.655197	−1.486594	−0.170219
44	8	0	−9.160895	−1.145796	1.112415
45	1	0	−0.439215	2.071199	1.737645
46	1	0	8.103630	−2.190905	1.453529
47	1	0	6.783693	−0.552323	−2.312525
48	1	0	0.696290	2.621113	−1.999938
49	1	0	0.462570	0.933516	−1.679423
50	1	0	5.239458	−1.025864	3.111721
51	1	0	9.614689	−1.118772	−1.082382
52	1	0	9.236327	−2.811749	−0.679425
53	1	0	8.697529	−2.139692	−2.217377
54	1	0	2.561081	1.129143	2.792685
55	1	0	1.496109	−1.060042	−1.254555
56	1	0	2.770292	−1.244458	−2.476376
57	1	0	3.028645	−1.832235	−0.848881
58	1	0	3.446311	1.041220	−3.493154
59	1	0	−1.193752	3.256207	−0.955228
60	1	0	−1.088731	4.008382	0.629022
61	1	0	−7.152715	0.762005	0.132095
62	1	0	−6.799003	−3.494331	−0.478918
63	1	0	−3.298879	−3.351384	−0.409218
64	1	0	−0.688612	−1.342044	0.545288
65	1	0	−4.010948	2.448278	1.868133
66	1	0	−3.778543	0.786293	−2.370360

67	1	0	−4.043179	2.498911	−2.121963
68	1	0	−2.389671	1.872999	−2.304790
69	1	0	−8.981550	−2.504233	−0.406558
70	1	0	−9.092337	−0.813335	−0.914555
71	1	0	−8.525637	−1.471851	1.772937

Table S7. Cartesian coordinates for the low-energy optimized conformer of **1**f at B3LYP/6–311+g (d,p) level of theory in CH_3CN.

**Conformer of 1f**			**Standard Orientation (Å)**		
Number	Atom	Type	X	Y	Z
1	6	0	−7.392929	−1.594754	−0.821999
2	6	0	−7.567762	−1.483609	0.563837
3	6	0	−6.581414	−0.843733	1.325640
4	6	0	−5.421062	−0.358271	0.720261
5	6	0	−5.252970	−0.471425	−0.656546
6	6	0	−6.244377	−1.088709	−1.427064
7	8	0	−4.516879	0.247375	1.545893
8	6	0	−3.143480	0.269515	1.082597
9	6	0	−3.026915	0.662832	−0.366555
10	6	0	−4.058059	0.113588	−1.278637
11	6	0	−2.465781	1.362561	1.960292
12	6	0	−1.045183	1.671475	1.501174
13	6	0	−0.990146	2.122482	0.025018
14	6	0	−2.076118	1.495304	−0.842017
15	8	0	−6.161358	−1.224518	−2.786599
16	6	0	−8.824255	−1.985856	1.210655
17	8	0	−3.943839	0.148048	−2.502417
18	8	0	−2.031216	1.904791	−2.164966
19	6	0	−2.535313	−1.123800	1.344192
20	1	0	−3.062770	2.284766	1.927445
21	8	0	−2.417048	0.977896	3.340742
22	6	0	0.452706	1.983012	−0.582012
23	1	0	−1.232505	3.196364	0.023479
24	6	0	1.371994	3.079140	0.012891
25	6	0	2.847416	2.822811	−0.279395
26	6	0	3.316467	1.445402	0.272635
27	6	0	2.348337	0.361346	−0.130207
28	6	0	1.067152	0.586272	−0.490830
29	8	0	4.641467	1.253599	−0.275437
30	6	0	5.142232	−0.016405	−0.216330
31	6	0	4.353150	−1.158037	−0.124919
32	6	0	2.892045	−1.018122	−0.129201
33	6	0	6.532425	−0.131920	−0.282270

34	6	0	7.149125	−1.390914	−0.234604
35	6	0	6.348825	−2.534540	−0.118673
36	6	0	4.961002	−2.418259	−0.074385
37	8	0	4.255801	−3.588005	0.015471
38	8	0	2.166016	−2.010588	−0.146895
39	8	0	0.204251	−0.436631	−0.848997
40	8	0	3.050338	2.906622	−1.697983
41	1	0	3.454628	3.626363	0.154234
42	6	0	3.430643	1.516648	1.810451
43	6	0	8.648107	−1.499569	−0.282801
44	8	0	9.209391	−1.022964	0.931267
45	1	0	0.388314	2.199133	−1.658244
46	1	0	−8.154387	−2.067036	−1.439587
47	1	0	−6.712239	−0.729437	2.400125
48	1	0	−0.628238	2.456062	2.145938
49	1	0	−0.408756	0.798321	1.686483
50	1	0	−5.340845	−0.777710	−3.090673
51	1	0	−9.221972	−2.852765	0.672237
52	1	0	−8.632619	−2.303333	2.241257
53	1	0	−9.582166	−1.196429	1.219368
54	1	0	−2.646784	1.344956	−2.688020
55	1	0	−2.698001	−1.434799	2.382922
56	1	0	−3.007825	−1.892795	0.722993
57	1	0	−1.461642	−1.155829	1.139582
58	1	0	−3.334243	0.765475	3.596240
59	1	0	1.229539	3.169910	1.093723
60	1	0	1.077354	4.043702	−0.422086
61	1	0	7.145132	0.766302	−0.361650
62	1	0	6.798695	−3.525700	−0.075461
63	1	0	3.297271	−3.375893	−0.025449
64	1	0	0.665102	−1.297575	−0.744000
65	1	0	3.953954	2.579560	−1.870866
66	1	0	3.850297	0.595707	2.230638
67	1	0	4.111269	2.322768	2.108294
68	1	0	2.463318	1.685025	2.294187
69	1	0	9.049735	−0.911648	−1.115010
70	1	0	8.968189	−2.536754	−0.425391
71	1	0	8.601625	−1.268502	1.649465

Table S8. Cartesian coordinates for the low-energy optimized conformer of **1**g at B3LYP/6–311+g (d,p) level of theory in CH_3CN.

**Conformer of 1g**			**Standard Orientation (Å)**		
Number	Atom	Type	X	Y	Z
1	6	0	6.856004	−2.758031	−0.235102
2	6	0	7.667208	−1.649306	0.033181
3	6	0	7.067938	−0.401207	0.247182
4	6	0	5.682186	−0.255341	0.155168
5	6	0	4.880772	−1.361487	−0.111000
6	6	0	5.470775	−2.615379	−0.304348
7	8	0	5.189301	1.000298	0.371476
8	6	0	3.901460	1.294048	−0.220266
9	6	0	2.904015	0.188311	0.039422
10	6	0	3.422359	−1.192025	−0.127566
11	6	0	3.422770	2.611820	0.465344
12	6	0	1.950674	2.896943	0.178221
13	6	0	1.035965	1.766906	0.690611
14	6	0	1.626321	0.384589	0.430027
15	8	0	4.750627	−3.751874	−0.556069
16	6	0	9.154515	−1.808293	0.154257
17	8	0	2.675843	−2.161744	−0.252104
18	8	0	0.749088	−0.660034	0.663867
19	6	0	4.097661	1.505065	−1.738361
20	1	0	3.584708	2.536418	1.548773
21	8	0	4.189362	3.738283	0.019846
22	6	0	−0.427514	1.992866	0.165807
23	1	0	1.024939	1.872936	1.784738
24	6	0	−0.563685	1.660894	−1.340999
25	6	0	−2.017839	1.482383	−1.767056
26	6	0	−2.747288	0.387262	−0.934166
27	6	0	−2.520157	0.615957	0.538009
28	6	0	−1.488359	1.327960	1.036642
29	8	0	−4.140602	0.510018	−1.310931
30	6	0	−5.040885	−0.113503	−0.495350
31	6	0	−4.805270	−0.385632	0.848540
32	6	0	−3.535539	0.038642	1.449940
33	6	0	−6.268092	−0.447738	−1.071975
34	6	0	−7.261166	−1.096740	−0.321498
35	6	0	−7.014202	−1.367075	1.032442
36	6	0	−5.797786	−1.010928	1.611968
37	8	0	−5.651565	−1.297451	2.942353
38	8	0	−3.348357	−0.075286	2.661089

39	8	0	−1.329905	1.572815	2.391939
40	8	0	−2.678976	2.753132	−1.643656
41	1	0	−2.060869	1.229860	−2.833709
42	6	0	−2.251981	−1.010359	−1.360614
43	6	0	−8.597751	−1.444879	−0.922782
44	8	0	−8.459098	−1.764606	−2.300307
45	1	0	−0.635261	3.070047	0.267408
46	1	0	7.295831	−3.742637	−0.382643
47	1	0	7.679668	0.469801	0.476354
48	1	0	1.662563	3.843811	0.654214
49	1	0	1.814418	3.069186	−0.895864
50	1	0	3.796116	−3.524945	−0.520239
51	1	0	9.514837	−2.611743	−0.496623
52	1	0	9.670317	−0.891085	−0.146443
53	1	0	9.422932	−2.044106	1.189058
54	1	0	1.191513	−1.508163	0.438597
55	1	0	3.174570	1.824572	−2.233133
56	1	0	4.875883	2.251155	−1.935595
57	1	0	4.430688	0.589073	−2.239138
58	1	0	3.960465	4.491843	0.591225
59	1	0	−0.003168	0.758420	−1.604149
60	1	0	−0.121321	2.480653	−1.920890
61	1	0	−6.448221	−0.217243	−2.120843
62	1	0	−7.769923	−1.849738	1.647217
63	1	0	−4.779925	−0.949313	3.236602
64	1	0	−1.971437	1.015666	2.886191
65	1	0	−3.632093	2.575171	−1.752187
66	1	0	−2.790647	−1.808507	−0.837826
67	1	0	−2.433280	−1.171684	−2.429796
68	1	0	−1.186217	−1.154104	−1.168053
69	1	0	−9.278595	−0.592533	−0.822978
70	1	0	−9.031131	−2.314296	−0.418007
71	1	0	−9.341750	−2.021269	−2.616353

Table S9. Cartesian coordinates for the low-energy optimized conformer of **1**h at B3LYP/6–311+g (d,p) level of theory in CH_3CN.

**Conformer of 1h**			**Standard Orientation (Å)**		
Number	Atom	Type	X	Y	Z
1	6	0	6.907165	−2.683360	−0.376023
2	6	0	7.703280	−1.579743	−0.045872
3	6	0	7.086341	−0.353214	0.233408
4	6	0	5.699363	−0.221194	0.146067
5	6	0	4.913717	−1.321836	−0.181530

6	6	0	5.521417	−2.555292	−0.441095
7	8	0	5.189828	1.014501	0.428536
8	6	0	3.898054	1.321848	−0.149969
9	6	0	2.915628	0.190628	0.046925
10	6	0	3.453541	−1.171537	−0.192871
11	6	0	3.401296	2.594388	0.603844
12	6	0	1.925697	2.874438	0.329201
13	6	0	1.024559	1.706988	0.777341
14	6	0	1.635111	0.348895	0.444077
15	8	0	4.816933	−3.685949	−0.755199
16	6	0	9.192221	−1.725936	0.069871
17	8	0	2.719585	−2.142920	−0.371904
18	8	0	0.771399	−0.718589	0.618822
19	6	0	4.094562	1.617441	−1.653948
20	1	0	3.561709	2.462589	1.682819
21	8	0	4.154078	3.753184	0.222321
22	6	0	−0.439865	1.941460	0.261684
23	1	0	1.010595	1.752802	1.876148
24	6	0	−0.569202	1.688592	−1.261137
25	6	0	−2.019714	1.514630	−1.699659
26	6	0	−2.736916	0.366426	−0.928516
27	6	0	−2.515730	0.520303	0.554234
28	6	0	−1.493656	1.217066	1.093109
29	8	0	−4.129121	0.489050	−1.305766
30	6	0	−5.027922	−0.176277	−0.520745
31	6	0	−4.792417	−0.517836	0.806323
32	6	0	−3.524858	−0.117820	1.430611
33	6	0	−6.254009	−0.486283	−1.115633
34	6	0	−7.254014	−1.155838	−0.393175
35	6	0	−6.994256	−1.520834	0.935758
36	6	0	−5.778437	−1.192625	1.533141
37	8	0	−5.624441	−1.562839	2.842071
38	8	0	−3.334924	−0.301800	2.632966
39	8	0	−1.340365	1.389349	2.460012
40	8	0	−2.697180	2.767796	−1.509845
41	1	0	−2.058393	1.319982	−2.778952
42	6	0	−2.221839	−0.998722	−1.426381
43	6	0	−8.567041	−1.539665	−1.026491
44	8	0	−8.895404	−0.658518	−2.091349
45	1	0	−0.662837	3.008310	0.420654
46	1	0	7.361437	−3.652148	−0.575911
47	1	0	7.686201	0.511845	0.511230
48	1	0	1.623586	3.790714	0.854091

49	1	0	1.788645	3.103052	−0.733928
50	1	0	3.858914	−3.474180	−0.709652
51	1	0	9.461755	−2.012002	1.091364
52	1	0	9.564715	−2.487244	−0.621767
53	1	0	9.697226	−0.786379	−0.178748
54	1	0	1.227068	−1.547902	0.351416
55	1	0	3.168799	1.951587	−2.133011
56	1	0	4.863260	2.382723	−1.808099
57	1	0	4.441386	0.734582	−2.202548
58	1	0	3.912009	4.472537	0.832166
59	1	0	0.004167	0.809406	−1.570081
60	1	0	−0.136881	2.543591	−1.794637
61	1	0	−6.432527	−0.202998	−2.151901
62	1	0	−7.743625	−2.051525	1.519870
63	1	0	−4.760095	−1.218879	3.158015
64	1	0	−1.976639	0.798729	2.921568
65	1	0	−3.649481	2.584869	−1.627625
66	1	0	−2.752356	−1.831310	−0.949849
67	1	0	−2.395494	−1.105448	−2.503399
68	1	0	−1.154241	−1.139109	−1.235660
69	1	0	−9.373707	−1.487740	−0.287979
70	1	0	−8.499868	−2.559075	−1.420719
71	1	0	−9.770879	−0.928032	−2.415470

Table S10. Cartesian coordinates for the low-energy optimized conformer of **1**i at B3LYP/6–311+g (d,p) level of theory in CH_3CN.

**Conformer of 1i**			**Standard Orientation (Å)**		
Number	Atom	Type	X	Y	Z
1	6	0	6.927965	−2.654575	−0.388355
2	6	0	7.715824	−1.554457	−0.027939
3	6	0	7.091177	−0.335208	0.269858
4	6	0	5.703702	−0.207665	0.171242
5	6	0	4.926000	−1.305753	−0.185472
6	6	0	5.541694	−2.530226	−0.463683
7	8	0	5.185579	1.019108	0.473833
8	6	0	3.899668	1.333196	−0.110629
9	6	0	2.919939	0.194818	0.054025
10	6	0	3.465689	−1.161140	−0.207900
11	6	0	3.388781	2.587848	0.663415
12	6	0	1.914187	2.866919	0.379640
13	6	0	1.014722	1.686594	0.795935
14	6	0	1.634669	0.338763	0.441627
15	8	0	4.846266	−3.658200	−0.808786

16	6	0	9.203639	−1.695613	0.099431
17	8	0	2.738173	−2.130777	−0.414331
18	8	0	0.773837	−0.736543	0.585850
19	6	0	4.108410	1.659305	−1.607171
20	1	0	3.540499	2.434944	1.741199
21	8	0	4.139256	3.757749	0.311240
22	6	0	−0.445763	1.924884	0.272501
23	1	0	0.991235	1.710616	1.895716
24	6	0	−0.559802	1.703715	−1.256893
25	6	0	−2.005172	1.528936	−1.712852
26	6	0	−2.720874	0.360803	−0.972854
27	6	0	−2.515284	0.484655	0.515397
28	6	0	−1.503366	1.177365	1.078649
29	8	0	−4.112025	0.481079	−1.359030
30	6	0	−5.010975	−0.209670	−0.597620
31	6	0	−4.785685	−0.576050	0.725630
32	6	0	−3.528205	−0.177755	1.369724
33	6	0	−6.228217	−0.516720	−1.209554
34	6	0	−7.221012	−1.222558	−0.512730
35	6	0	−6.983154	−1.596808	0.816004
36	6	0	−5.775893	−1.269427	1.430942
37	8	0	−5.635824	−1.654134	2.736850
38	8	0	−3.349458	−0.381938	2.569442
39	8	0	−1.364354	1.322776	2.450107
40	8	0	−2.692956	2.774163	−1.502392
41	1	0	−2.031727	1.356514	−2.795487
42	6	0	−2.192007	−0.990583	−1.494224
43	6	0	−8.527485	−1.549617	−1.183551
44	8	0	−9.261840	−0.358715	−1.429904
45	1	0	−0.676271	2.987603	0.451056
46	1	0	7.388372	−3.616829	−0.603373
47	1	0	7.684300	0.525508	0.571103
48	1	0	1.604309	3.771964	0.920094
49	1	0	1.787244	3.116442	−0.679175
50	1	0	3.886272	−3.451337	−0.768053
51	1	0	9.464542	−2.002012	1.117148
52	1	0	9.586947	−2.442270	−0.604478
53	1	0	9.707441	−0.749241	−0.125244
54	1	0	1.236612	−1.558286	0.307580
55	1	0	3.185823	1.998943	−2.087986
56	1	0	4.875704	2.431215	−1.737925
57	1	0	4.464599	0.788533	−2.169753
58	1	0	3.893186	4.461866	0.935870

59	1	0	0.021699	0.835065	−1.578829
60	1	0	−0.128727	2.573197	−1.768405
61	1	0	−6.406865	−0.210044	−2.239433
62	1	0	−7.736281	−2.137322	1.385135
63	1	0	−4.772741	−1.316588	3.066419
64	1	0	−1.997520	0.717487	2.894220
65	1	0	−3.640760	2.590600	−1.645647
66	1	0	−2.719590	−1.836172	−1.038680
67	1	0	−2.356882	−1.075775	−2.573926
68	1	0	−1.125063	−1.127036	−1.297927
69	1	0	−9.142455	−2.207645	−0.561425
70	1	0	−8.351139	−2.061187	−2.135897
71	1	0	−9.085023	0.250843	−0.692498

Table S11. Cartesian coordinates for the low-energy optimized conformer of **1**j at B3LYP/6–311+g (d,p) level of theory in CH_3CN.

**Conformer of 1j**			**Standard Orientation (Å)**		
Number	Atom	Type	X	Y	Z
1	6	0	6.826985	−2.792852	−0.187271
2	6	0	7.645764	−1.688204	0.078917
3	6	0	7.054582	−0.432522	0.279451
4	6	0	5.670666	−0.277174	0.177617
5	6	0	4.862156	−1.379268	−0.084964
6	6	0	5.443973	−2.637966	−0.265568
7	8	0	5.187481	0.983679	0.381114
8	6	0	3.905328	1.283242	−0.219875
9	6	0	2.897421	0.188164	0.041774
10	6	0	3.406164	−1.197635	−0.112474
11	6	0	3.432403	2.609963	0.452559
12	6	0	1.964731	2.904132	0.153495
13	6	0	1.037840	1.785403	0.669209
14	6	0	1.619377	0.397088	0.422595
15	8	0	4.716980	−3.771620	−0.514438
16	6	0	9.130880	−1.857809	0.209893
17	8	0	2.652177	−2.162385	−0.235520
18	8	0	0.731814	−0.639617	0.657356
19	6	0	4.112606	1.482133	−1.739364
20	1	0	3.588095	2.541202	1.537062
21	8	0	4.210867	3.726657	0.002415
22	6	0	−0.420541	2.019560	0.134166
23	1	0	1.021748	1.899910	1.762338
24	6	0	−0.550974	1.677914	−1.370429
25	6	0	−2.003102	1.505109	−1.804511

26	6	0	−2.744057	0.420470	−0.968460
27	6	0	−2.524727	0.659160	0.503973
28	6	0	−1.490988	1.367805	1.003271
29	8	0	−4.134636	0.545399	−1.357211
30	6	0	−5.046370	−0.055700	−0.537268
31	6	0	−4.820768	−0.319292	0.809964
32	6	0	−3.548235	0.094282	1.414406
33	6	0	−6.276180	−0.379047	−1.116256
34	6	0	−7.284249	−0.992480	−0.356797
35	6	0	−7.041797	−1.274938	0.992688
36	6	0	−5.821539	−0.931629	1.572818
37	8	0	−5.679104	−1.220834	2.903284
38	8	0	−3.366497	−0.018435	2.625271
39	8	0	−1.339117	1.620492	2.357610
40	8	0	−2.656581	2.780602	−1.692924
41	1	0	−2.041862	1.246019	−2.869480
42	6	0	−2.254070	−0.982915	−1.380400
43	6	0	−8.595494	−1.356756	−0.998764
44	8	0	−8.408306	−2.424752	−1.915544
45	1	0	−0.622130	3.098120	0.226946
46	1	0	7.260626	−3.780838	−0.325086
47	1	0	7.672088	0.433891	0.506121
48	1	0	1.681384	3.857152	0.619522
49	1	0	1.835835	3.069172	−0.922159
50	1	0	3.763647	−3.536684	−0.485478
51	1	0	9.488773	−2.669028	−0.432662
52	1	0	9.656115	−0.946597	−0.093203
53	1	0	9.390228	−2.087699	1.248374
54	1	0	1.171303	−1.492980	0.444717
55	1	0	4.898313	2.218541	−1.936977
56	1	0	4.442359	0.558851	−2.230212
57	1	0	3.194980	1.805099	−2.241476
58	1	0	3.987194	4.485437	0.568595
59	1	0	0.006552	0.770549	−1.624734
60	1	0	−0.100161	2.490779	−1.953691
61	1	0	−6.448337	−0.162181	−2.168922
62	1	0	−7.803299	−1.752434	1.606496
63	1	0	−4.808564	−0.875163	3.202418
64	1	0	−1.984868	1.070556	2.852830
65	1	0	−3.610931	2.611024	−1.812968
66	1	0	−2.801002	−1.774540	−0.855951
67	1	0	−2.427834	−1.151547	−2.449962
68	1	0	−1.189700	−1.131379	−1.178460

69	1	0	−9.012285	−0.497782	−1.534533
70	1	0	−9.330811	−1.670624	−0.250997
71	1	0	−7.724776	−3.009756	−1.546137

Table S12. Gibbs free energy and Boltzmann population of low energy of 5*S*,7*R*,10a*R*,5'*S*,7'*R*,10a'*R*-**2** in CH_3CN.

Conformers of 5*S*,7*R*,10a*R*,5'*S*,7'*R*,10a'*R*-2	Δ*G*（kcal/mol）	P
**2**a	0	0.288
**2**b	0.47	0.239
**2**c	0.49	0.237
**2**d	0.58	0.228

Table S13. Cartesian coordinates for the low-energy optimized conformer of **2**a at B3LYP/6–311+g (d,p) level of theory in CH_3CN.

**Conformer of 2a**			**Standard Orientation (Å)**		
Number	Atom	Type	X	Y	Z
1	6	0	6.494229	−2.684530	−0.787475
2	6	0	7.301673	−1.816132	−0.043099
3	6	0	6.755296	−0.615927	0.432791
4	6	0	5.412465	−0.307151	0.201518
5	6	0	4.615423	−1.173937	−0.539666
6	6	0	5.161147	−2.363448	−1.036370
7	8	0	4.972448	0.881461	0.711646
8	6	0	3.545952	0.985425	0.945657
9	6	0	2.728444	0.455270	−0.206733
10	6	0	3.228594	−0.791443	−0.832387
11	6	0	3.286536	2.507515	1.136203
12	6	0	1.798103	2.836561	1.157346
13	6	0	1.074203	2.369973	−0.131363
14	6	0	1.620832	1.058528	−0.689397
15	8	0	4.448267	−3.259826	−1.785307
16	8	0	2.532475	−1.477250	−1.581285
17	8	0	0.906714	0.555081	−1.764910
18	6	0	3.215577	0.232464	2.249769
19	6	0	8.749539	−2.137347	0.186754
20	6	0	−6.111143	−2.369567	0.988859
21	6	0	−6.886605	−1.509354	0.196932
22	6	0	−6.284181	−0.357753	−0.334074
23	6	0	−4.931639	−0.091739	−0.114319
24	6	0	−4.171774	−0.952110	0.672443
25	6	0	−4.766755	−2.089948	1.226429
26	8	0	−4.440775	1.050869	−0.681039

27	6	0	−3.012656	1.078690	−0.922055
28	6	0	−2.216096	0.574809	0.256378
29	6	0	−2.769807	−0.613612	0.948260
30	6	0	−2.687422	2.576265	−1.192917
31	6	0	−1.185372	2.837549	−1.230496
32	6	0	−0.480508	2.409365	0.080988
33	6	0	−1.083163	1.153665	0.708225
34	8	0	−4.093325	−2.975914	2.024275
35	8	0	−2.105640	−1.286625	1.734829
36	8	0	−0.389437	0.677422	1.809338
37	6	0	−2.719127	0.244408	−2.185300
38	6	0	−8.349481	−1.797831	−0.011879
39	8	0	3.902079	3.270231	0.084816
40	8	0	−3.266812	3.419748	−0.182823
41	8	0	−8.771291	−1.337519	−1.288346
42	1	0	−0.688130	3.217822	0.799874
43	1	0	1.315258	3.129751	−0.891071
44	1	0	−3.141741	2.892961	−2.139344
45	1	0	3.755512	2.853874	2.065858
46	1	0	6.900917	−3.611072	−1.186993
47	1	0	7.374663	0.078742	0.995988
48	1	0	1.342777	2.403377	2.052459
49	1	0	1.668661	3.923745	1.243656
50	1	0	3.549326	−2.891601	−1.933365
51	1	0	1.278611	−0.323218	−2.002776
52	1	0	2.159986	0.312505	2.524913
53	1	0	3.453452	−0.835173	2.176156
54	1	0	3.817450	0.619434	3.079854
55	1	0	9.098531	−1.710282	1.133002
56	1	0	8.905350	−3.219457	0.241554
57	1	0	9.356033	−1.732320	−0.629458
58	1	0	−6.551971	−3.260739	1.432206
59	1	0	−6.874383	0.329299	−0.939617
60	1	0	−0.750994	2.337855	−2.100540
61	1	0	−1.008181	3.912013	−1.374135
62	1	0	−3.178699	−2.639247	2.151752
63	1	0	−0.799971	−0.169491	2.092481
64	1	0	−1.661536	0.265001	−2.464535
65	1	0	−3.001126	−0.806329	−2.054819
66	1	0	−3.305909	0.613102	−3.033980
67	1	0	−8.938070	−1.293730	0.760142
68	1	0	−8.539294	−2.875261	0.034062
69	1	0	4.806415	2.917119	−0.015032

70	1	0	−4.188818	3.117113	−0.074173
71	1	0	−9.705445	−1.589161	−1.379792

Table S14. Cartesian coordinates for the low-energy optimized conformer of **2**b at B3LYP/6–311+g (d,p) level of theory in CH_3CN.

**Conformer of 2b**			**Standard Orientation (Å)**		
Number	Atom	Type	X	Y	Z
1	6	0	6.538642	−2.625115	−0.789089
2	6	0	7.328251	−1.765583	−0.015038
3	6	0	6.764672	−0.581973	0.479878
4	6	0	5.421865	−0.280589	0.236913
5	6	0	4.642720	−1.138260	−0.534302
6	6	0	5.206967	−2.310758	−1.048841
7	8	0	4.964178	0.892536	0.766193
8	6	0	3.533333	0.978225	0.982691
9	6	0	2.735719	0.465912	−0.190846
10	6	0	3.256310	−0.762262	−0.837406
11	6	0	3.257123	2.494105	1.202945
12	6	0	1.765045	2.808107	1.211776
13	6	0	1.062965	2.364002	−0.095537
14	6	0	1.630142	1.069295	−0.676059
15	8	0	4.512114	−3.198241	−1.827283
16	8	0	2.577238	−1.436587	−1.610505
17	8	0	0.934065	0.583231	−1.771805
18	6	0	3.193012	0.193436	2.266508
19	6	0	8.775694	−2.079227	0.227560
20	6	0	−6.096477	−2.457036	0.841538
21	6	0	−6.876851	−1.572062	0.084988
22	6	0	−6.270953	−0.419284	−0.440621
23	6	0	−4.920649	−0.150174	−0.205479
24	6	0	−4.162950	−1.020809	0.571716
25	6	0	−4.754226	−2.176725	1.093041
26	8	0	−4.431558	1.002039	−0.750730
27	6	0	−3.000650	1.050438	−0.968358
28	6	0	−2.214890	0.528675	0.209256
29	6	0	−2.765452	−0.679340	0.866607
30	6	0	−2.686193	2.557035	−1.202130
31	6	0	−1.187360	2.833399	−1.213929
32	6	0	−0.494944	2.383257	0.097055
33	6	0	−1.092814	1.107819	0.687613
34	8	0	−4.077688	−3.081825	1.865434
35	8	0	−2.101870	−1.368718	1.640789

36	8	0	−0.408065	0.613177	1.786790
37	6	0	−2.680091	0.247127	−2.245335
38	6	0	−8.319226	−1.903365	−0.194517
39	8	0	3.879035	3.285252	0.176670
40	8	0	−3.286841	3.371328	−0.180930
41	8	0	−9.079212	−0.721939	−0.409371
42	1	0	−0.719083	3.172644	0.830561
43	1	0	1.306378	3.141676	−0.836143
44	1	0	−3.131963	2.890668	−2.146905
45	1	0	3.709278	2.823836	2.145812
46	1	0	6.959435	−3.539161	−1.203165
47	1	0	7.369418	0.106641	1.066575
48	1	0	1.302744	2.351046	2.091123
49	1	0	1.624125	3.891631	1.319966
50	1	0	3.611950	−2.833109	−1.978705
51	1	0	1.318701	−0.285452	−2.023438
52	1	0	2.134061	0.258864	2.528601
53	1	0	3.441817	−0.868864	2.172993
54	1	0	3.780474	0.568934	3.113169
55	1	0	9.107902	−1.669880	1.187310
56	1	0	8.940904	−3.160272	0.261837
57	1	0	9.389154	−1.651289	−0.570888
58	1	0	−6.530877	−3.365479	1.253229
59	1	0	−6.858006	0.274855	−1.039505
60	1	0	−0.737329	2.357249	−2.089333
61	1	0	−1.019052	3.912215	−1.332315
62	1	0	−3.169351	−2.738455	2.016462
63	1	0	−0.815447	−0.243395	2.046137
64	1	0	−1.619554	0.281611	−2.507267
65	1	0	−2.956871	−0.808985	−2.141488
66	1	0	−3.257157	0.629113	−3.095004
67	1	0	−8.760033	−2.434286	0.656129
68	1	0	−8.385247	−2.531773	−1.087856
69	1	0	4.788288	2.943378	0.082298
70	1	0	−4.206620	3.057234	−0.086923
71	1	0	−10.001602	−1.002848	−0.530866

Table S15. Cartesian coordinates for the low-energy optimized conformer of **2**c at B3LYP/6–311+g (d,p) level of theory in CH_3CN.

**Conformer of 2c**			**Standard Orientation (Å)**		
Number	Atom	Type	X	Y	Z
1	6	0	−6.541433	−2.624778	0.760211
2	6	0	−7.323310	−1.773280	−0.030003

3	6	0	−6.755078	−0.593517	−0.529160
4	6	0	−5.415657	−0.287792	−0.274016
5	6	0	−4.644669	−1.137558	0.514342
6	6	0	−5.212468	−2.306883	1.032549
7	8	0	−4.953044	0.881342	−0.808412
8	6	0	−3.520474	0.966734	−1.008965
9	6	0	−2.735971	0.466470	0.177635
10	6	0	−3.261895	−0.756666	0.829366
11	6	0	−3.242881	2.481528	−1.240443
12	6	0	−1.751550	2.796602	−1.233576
13	6	0	−1.064168	2.364862	0.086724
14	6	0	−1.636294	1.075458	0.670720
15	8	0	−4.525847	−3.185329	1.826258
16	8	0	−2.591010	−1.424194	1.615899
17	8	0	−0.952633	0.598910	1.778166
18	6	0	−3.163926	0.171276	−2.281984
19	6	0	−8.767127	−2.090078	−0.285943
20	6	0	6.110999	−2.456309	−0.711647
21	6	0	6.871404	−1.572145	0.064122
22	6	0	6.265568	−0.407151	0.558357
23	6	0	4.918986	−0.141313	0.301598
24	6	0	4.172780	−1.018780	−0.478479
25	6	0	4.773407	−2.176513	−0.986023
26	8	0	4.423232	1.018755	0.826475
27	6	0	2.988559	1.067698	1.024653
28	6	0	2.219569	0.533873	−0.158227
29	6	0	2.779673	−0.680362	−0.797572
30	6	0	2.669675	2.575920	1.239349
31	6	0	1.169805	2.850705	1.228538
32	6	0	0.495609	2.387016	−0.087217
33	6	0	1.103797	1.106770	−0.658624
34	8	0	4.112310	−3.082577	−1.770531
35	8	0	2.129388	−1.374371	−1.577989
36	8	0	0.434815	0.601585	−1.761935
37	6	0	2.651923	0.275859	2.304561
38	6	0	8.322711	−1.847372	0.349681
39	8	0	−3.876559	3.280375	−0.227451
40	8	0	3.282910	3.381326	0.219007
41	8	0	9.142263	−0.911023	−0.333751
42	1	0	0.728116	3.169761	−0.825152
43	1	0	−1.318466	3.149495	0.816128
44	1	0	3.101564	2.918743	2.187664
45	1	0	−3.685299	2.802164	−2.190463

46	1	0	−6.966478	−3.535487	1.176929
47	1	0	−7.354701	0.089820	−1.127962
48	1	0	−1.277548	2.332108	−2.102871
49	1	0	−1.609360	3.879254	−1.350965
50	1	0	−3.628324	−2.819606	1.985193
51	1	0	−1.339997	−0.267549	2.034548
52	1	0	−2.101903	0.235230	−2.532388
53	1	0	−3.412990	−0.891129	−2.181972
54	1	0	−3.742224	0.538323	−3.138326
55	1	0	−9.089805	−1.689030	−1.253457
56	1	0	−8.931067	−3.171667	−0.313112
57	1	0	−9.390096	−1.655825	0.501259
58	1	0	6.555978	−3.363504	−1.117537
59	1	0	6.849105	0.294611	1.153568
60	1	0	0.709112	2.382276	2.102761
61	1	0	0.998180	3.930283	1.333950
62	1	0	3.202796	−2.745960	−1.931025
63	1	0	0.843434	−0.257815	−2.007455
64	1	0	1.588187	0.313022	2.553773
65	1	0	2.930346	−0.780138	2.214770
66	1	0	3.218826	0.667042	3.157895
67	1	0	8.608662	−2.853617	0.024047
68	1	0	8.521799	−1.777344	1.424212
69	1	0	−4.788113	2.939973	−0.140925
70	1	0	4.207574	3.077880	0.149736
71	1	0	8.700707	−0.691362	−1.171871

Table S16. Cartesian coordinates for the low-energy optimized conformer of **2**d at B3LYP/6–311+g (d,p) level of theory in CH_3CN.

**Conformer of 2d**			**Standard Orientation (Å)**		
Number	Atom	Type	X	Y	Z
1	6	0	6.454829	−2.738907	−0.743018
2	6	0	7.271573	−1.865052	−0.014337
3	6	0	6.737606	−0.651910	0.441983
4	6	0	5.398108	−0.334283	0.206812
5	6	0	4.591152	−1.206562	−0.519278
6	6	0	5.124168	−2.408415	−0.995464
7	8	0	4.969309	0.867119	0.695977
8	6	0	3.544850	0.988290	0.930856
9	6	0	2.719969	0.447324	−0.211411
10	6	0	3.206839	−0.815151	−0.816035
11	6	0	3.299742	2.517276	1.095874
12	6	0	1.814592	2.859813	1.113231

13	6	0	1.085457	2.378709	−0.166973
14	6	0	1.618533	1.052914	−0.703880
15	8	0	4.401274	−3.311243	−1.727987
16	8	0	2.503668	−1.506474	−1.551728
17	8	0	0.897300	0.537427	−1.768822
18	6	0	3.208879	0.260742	2.248142
19	6	0	8.716757	−2.196485	0.219409
20	6	0	−6.148578	−2.265445	1.045090
21	6	0	−6.916613	−1.397556	0.257602
22	6	0	−6.302163	−0.273508	−0.313853
23	6	0	−4.945028	−0.021133	−0.105504
24	6	0	−4.193477	−0.875326	0.695791
25	6	0	−4.798975	−1.999386	1.268170
26	8	0	−4.442843	1.101436	−0.699660
27	6	0	−3.012993	1.114646	−0.936254
28	6	0	−2.222208	0.623075	0.249696
29	6	0	−2.786749	−0.548772	0.960382
30	6	0	−2.673802	2.604544	−1.231814
31	6	0	−1.170276	2.851945	−1.273156
32	6	0	−0.469514	2.438038	0.045854
33	6	0	−1.083852	1.198933	0.692614
34	8	0	−4.129895	−2.884413	2.069379
35	8	0	−2.126593	−1.219733	1.753545
36	8	0	−0.394594	0.733423	1.800878
37	6	0	−2.725443	0.257661	−2.185646
38	6	0	−8.374860	−1.671454	0.005868
39	8	0	3.920418	3.255946	0.030121
40	8	0	−3.244930	3.469058	−0.234887
41	8	0	−8.570831	−2.062521	−1.344727
42	1	0	−0.667772	3.259311	0.750840
43	1	0	1.333094	3.122994	−0.939657
44	1	0	−3.125132	2.911619	−2.183114
45	1	0	3.773060	2.874423	2.018416
46	1	0	6.851050	−3.675681	−1.127207
47	1	0	7.364435	0.047190	0.992050
48	1	0	1.356043	2.445440	2.015080
49	1	0	1.694435	3.949478	1.181780
50	1	0	3.505875	−2.935876	−1.882030
51	1	0	1.263078	−0.346651	−1.993649
52	1	0	2.155305	0.356418	2.522366
53	1	0	3.436309	−0.809458	2.192467
54	1	0	3.815182	0.657629	3.071026
55	1	0	8.861539	−3.279499	0.292293

56	1	0	9.326087	−1.812061	−0.604741
57	1	0	9.071060	−1.756188	1.156592
58	1	0	−6.596648	−3.147204	1.498005
59	1	0	−6.886479	0.403835	−0.935818
60	1	0	−0.740270	2.333682	−2.135072
61	1	0	−0.982321	3.921884	−1.433724
62	1	0	−3.212560	−2.555075	2.195725
63	1	0	−0.811469	−0.105830	2.098159
64	1	0	−1.668403	0.262209	−2.462882
65	1	0	−3.019495	−0.787795	−2.039009
66	1	0	−3.307104	0.618255	−3.042257
67	1	0	−8.973481	−0.777244	0.207576
68	1	0	−8.747393	−2.472401	0.653415
69	1	0	4.822224	2.894225	−0.062895
70	1	0	−4.172433	3.183580	−0.125662
71	1	0	−7.787218	−2.568788	−1.619286
